# The novel microtubule-associated CAP-glycine protein Cgp1 governs growth, differentiation, and virulence of *Cryptococcus neoformans*

**DOI:** 10.1080/21505594.2017.1423189

**Published:** 2018-02-27

**Authors:** Li Li Wang, Kyung-Tae Lee, Kwang-Woo Jung, Dong-Gi Lee, Yong-Sun Bahn

**Affiliations:** a Department of Biotechnology, College of Life Science and Biotechnology, Yonsei University, Seoul, Republic of Korea; b Research Division for Biotechnology, Korea Atomic Energy Research Institute, Jeongeup, Republic of Korea

**Keywords:** Bik1, CAP-glycine, human fungal pathogen, meningoencephalitis, SPEC, Spc7, tubulin

## Abstract

Microtubules are involved in mechanical support, cytoplasmic organization, and several cellular processes by interacting with diverse microtubule-associated proteins such as plus-end tracking proteins, motor proteins, and tubulin-folding cofactors. A number of the cytoskeleton-associated proteins (CAPs) contain the CAP-glycine-rich (CAP-Gly) domain, which is evolutionarily conserved and generally considered to bind to α-tubulin to regulate the function of microtubules. However, there has been a dearth of research on CAP-Gly proteins in fungal pathogens, including *Cryptococcus neoformans*, which is a global cause of fatal meningoencephalitis in immunocompromised patients. In this study, we identified five CAP-Gly protein-encoding genes in *C. neoformans*. Among these, Cgp1 encoded by CNAG_06352 has a unique domain structure containing CAP-Gly, SPEC, and Spc7 domains that is not orthologous to CAPs in other eukaryotes. Supporting the role of Cgp1 in microtubule-related function, we demonstrate that deletion or overexpression of *CGP1* alters cellular susceptibility to thiabendazole, a microtubule destabilizer and that Cgp1 is co-localized with cytoplasmic microtubules. Related to the cellular function of microtubules, Cgp1 governs the maintenance of membrane stability and genotoxic stress responses. Deletion of *CGP1* also reduces production of melanin pigment and attenuates the virulence of *C. neoformans*. Furthermore, we demonstrate that Cgp1 uniquely regulates the sexual differentiation of *C. neoformans* with distinct roles in the early and late stage of mating. Domain analysis revealed that the CAP-Gly domain plays a major role in all Cgp1 functions examined. In conclusion, this novel CAP-Gly protein, Cgp1, has pleotropic roles in regulating growth, stress responses, differentiation, and virulence in *C. neoformans*.

## Introduction

Microtubules represent one of the cytoskeletal fiber systems in eukaryotes, playing essential roles in various cellular processes, including cell motility, intracellular trafficking, cell shape and polarity, and mitosis [1]. Microtubule-associated proteins (MAPs) regulate microtubule dynamics and activities by promoting tubulin polymerization and stabilizing microtubules, whereas phosphorylated MAPs destabilize microtubules [2]. Intracellular transport along microtubules is mediated by MAPs and the microtubule motor proteins including kinesins and dyneins [3]. Microtubule motors interact with cargo through by associating with adaptors or scaffolding proteins and carry it directionally along a cytoskeletal track [3,4].

The cytoskeleton-associated protein (CAP)-glycine-rich (CAP-Gly) domain, which consists of ∼80 amino acids, is an evolutionarily conserved protein-interaction module found in eukaryotes and is implicated in a number of diverse cellular processes. The conserved CAP-Gly domain has been identified in a number of CAPs, including CLIP-170 and dynactins [5]. Proteins containing the CAP-Gly domain interact with microtubules and MAPs by binding to the C-terminal EEY/F-COO- sequence motif present in α-tubulin and in microtubule end-binding proteins. This microtubule-binding activity of CAP-Gly proteins regulates microtubule organization and dynamics, chromosome segregation, cell polarity establishment, cell migration, and vesicle transport [6]. In addition, CAP-Gly proteins interact with other structural elements, such as end-binding homology domains, zinc-finger motifs, and proline-rich sequences [7–9]. Although several CAP-Gly proteins have been previously characterized in the model yeasts *Saccharomyces cerevisiae* and *Schizosaccharomyces pombe* [10-13], the function of CAP-Gly proteins have not been studied thoroughly in other fungal pathogens.

*C. neoformans* is a basidiomycetous fungal pathogen that is a global cause of a life-threatening meningoencephalitis. It is responsible for more than a million infections and 600,000 deaths annually [14]. To understand the molecular mechanism of pathogenesis by *C. neoformans*, we identified a novel CAP-Gly protein, which is encoded by CNAG_06352 in *Cryptococcus neoformans*, with a unique domain structure that we named Cgp1 (CAP-Gly protein 1). Despite the absence of homology to any known CAP-Gly proteins in eukaryotes, we demonstrate that Cgp1 is not only required for microtubule-associated cell functions, but also mediates diverse cellular functions, including growth under diverse environmental stresses, differentiation, and virulence in *C. neoformans*.

## Results

### Identification of the novel CAP-Gly protein Cgp1 in *C. neoformans*

**Figure 1.**
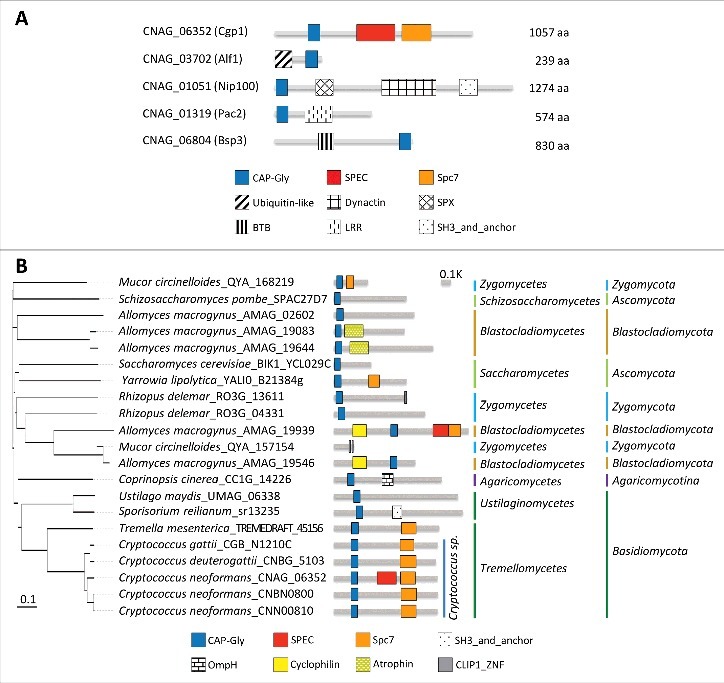
Structural and phylogenetic analyses of fungal CAP-Gly proteins. (A) Domain analysis was performed using the National Center for Biotechnology Information (NCBI) domain database (http://www.ncbi.nlm.nih.gov/Structure/cdd/wrpsb.cgi) and Pfam (http://pfam.xfam.org/). The domain abbreviations indicate the following: CAP-Gly (smart01052), Cytoskeleton-associated-protein-glycine-rich; SPEC (cd00176), Spectrin repeats, found in several proteins involved in cytoskeletal structure; Spc7 (smart00787), Spc7 kinetochore protein; Ubiquitin-like (pfam14560), Ubiquitin-like domain; Dynactin (pfam12455), Dynein associated protein; SPX (cl21499), Domain found in Syg1, Pho81, XPR1, and related proteins; BTB (smart00225), Broad-Complex, Tramtrack and Bric a brac; LRR (pfam13855), Leucine rich repeat; SH3_and_anchor (TIGR04211), SH3 domain protein; OmpH (smart00935), outer membrane protein (OmpH-like); Cyclophilin (cd00317), Cyclophilin-like peptidyl-prolyl cis-trans isomerase family protein; Atrophin (pfam03154), atrophin-1 family; CLIP1_ZNF (pfam16641), CLIP1 zinc knuckle. (A) CAP-Gly proteins in *C. neoformans* and their domain structure. (B) Phylogenetic analysis of Cgp1 orthologs in diverse fungal species.

We searched for CAP-Gly proteins in the *C. neoformans* var. *grubii* H99 genome by analyzing InterPro domain annotation (CAP Gly-rich domain: IPR000938) in FungiDB (http://fungiDB.org). As a result, five genes were predicted to encode CAP-Gly domains: CNAG_06352, CNAG_03702, CNAG_01051, CNAG_01319, and CNAG_06804. Next, we analyzed their protein domain structure using the Conserved Domain Search website (http://www.ncbi.nlm.nih.gov/Structure/cdd/wrpsb.cgi) (Fig. 1A). All five proteins contain a highly glycine-rich region, which is a signature for the CAP-Gly domain (Fig. S1).

CNAG_03702 is orthologous to Alf1, which is a tubulin folding cofactor in *S. cerevisiae* [15]. CNAG_01051 is orthologous to *S. cerevisiae* Nip100, which is a large subunit of the dynactin complex responsible for driving the movement of cargo along microtubules [16]. Donlin *et al.* also identified this gene as being *C. neoformans* Nip100 in a transcriptome study [17]. CNAG_01319 is orthologous to *S. cerevisiae* Pac2, which is a microtubule effector required for tubulin heterodimer formation [18]. CNAG_06804 is orthologous to BSP (binder sperm protein) proteins, which interact with the sperm membrane via choline phospholipids and play a role in sperm capacitation in mammals [19]. In *Cryptococcus* species, CNAG_06804 resides in the mating type locus and was previously named as Bsp3, however, its function remains unknown [20].

Among the *Cryptococcus* CAP-Gly proteins, CNAG_06352 was notable because it encodes a unique CAP-Gly protein of 1,057-amino acids that also contains SPEC and Spc7 domains. It is distantly related to mammalian CLIP1 and *S. cerevisiae* Bik1 but is structurally distinct because CLIP1 and Bik1 do not possess SPEC and Spc7 domains. Phylogenetic analysis revealed that the Spc7 domain is commonly observed in CNAG_06352 orthologs in *Tremellomycetes*, but not in *Ustilaginomycetes* (Fig. 1B), indicating that CNAG_06352 orthologs have divergently evolved in fungal species. Notably, *C. neoformans* serotype A H99 strain has an additional SPEC domain compared to other pathogenic *Cryptococcus* species complex (Fig. 1B). Therefore, we have named this novel CAP-Gly protein (CNAG_06352) Cgp1 (CAP-Gly protein 1) and consequently decided to further analyze the function of Cgp1 in *C. neoformans*.

### Cgp1 is involved in microtubule-associated functions in *C. neoformans*

**Figure 2.**
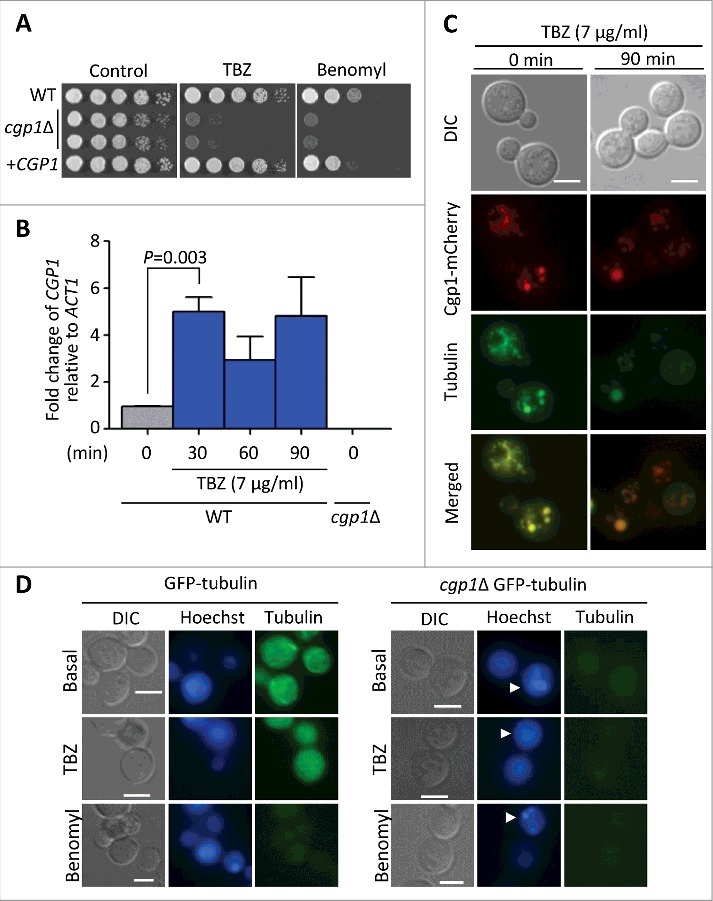
Cgp1 is involved in microtubule maintenance and is co-localized with tubulins. (A) Sensitivity test for *cgp1*Δ mutants to the microtubule destabilizer thiabendazole (TBZ) and benomyl. The WT (H99), *cgp1*Δ mutants (YSB1631 and YSB1632), and the *cgp1*Δ::*CGP1* complemented strain (+*CGP1*, YSB3332) were grown in 2 mL of liquid YPD medium, serially diluted 10-fold, and then spotted onto YPD agar medium containing the indicated concentration (7 µg/mL) of TBZ or (4 µg/mL) of benomyl, further incubated at 30°C and photographed daily for 2–4 days. (B) Expression of *CGP1* in response to TBZ assessed by qRT-PCR. The *C. neoformans* wild type strain (H99) was grown to the mid-logarithmic phase and then 7 µg/mL TBZ was added to the medium and incubation continued for 90 min, after which a portion of the cells was removed for total RNA isolation and qRT-PCR analysis. Three-independent experiments were performed. Actin was used to normalize the expression of *CGP1*. The *cgp1*Δ mutant (YSB1631) was used to confirm primer specificity for the *CGP1* gene. The error bar indicates SEM. The *P* value was calculated by Student's *t*-test after normalizing the data to the basal expression level. (C) Co-localization of Cgp1 and microtubules/tubulin in the cytoplasm. The *cgp1*Δ::*CGP1*-*mCherr*y-tagged strain grown to the mid-logarithmic phase was treated with TBZ (7 μg/mL) for 90 min or not-treated (0 min). Tubulins were stained with 0.5 μL of Tubulin Tracker Green. Cells were observed under a fluorescence microscope. The scale bars represent 10 μm. (D) GFP- tubulin strain (LK126) and *cgp1*Δ GFP-tubulin strain (YSB5536) were visualized by fluorescence microscope with Hoechst staining. Samples with or without the drugs (7 μg/mL TBZ or 4 μg/mL benomyl, treatment for 30 minutes) were observed by formalin fixation. White arrows indicate multinucleate cells.

To characterize the cellular functions of Cgp1, we constructed two independent *cgp1*Δ mutants in the *MAT*α serotype A *C. neoformans* H99 strain background (Fig. S2 and Table S1). To confirm the phenotype of *cgp1*Δ mutants, we also constructed a *cgp1*Δ*::CGP1* complemented strain by re-integrating the wild type *CGP1* gene into its native locus (Fig. S3). Deletion of *CGP1* caused minor growth defects, but not lethality, in *C. neoformans* (Fig. 2A).

CAP-Gly proteins generally interact with microtubules or other MAPs through the CAP-Gly domain. To address whether Cgp1 has microtubule-associated functions, we examined the susceptibility of *cgp1*Δ mutants to a microtubule-destabilizing agent, thiabendazole (TBZ), which affects the positioning of microtubules and inhibits their assembly [21], and benomyl, a fungicide that binds to the microtubules [22,23]. The *cgp1*Δ mutant strains exhibited a highly increased susceptibility to TBZ and benomyl (Fig. 2A). Verifying this result, the *cgp1*Δ::*CGP1* complemented strain restored TBZ and benomyl resistance to wild type levels (Fig. 2A). We also monitored the expression of *CGP1* in response to TBZ using quantitative reverse transcription PCR (qRT-PCR). The expression of *CGP1* was induced 30 min post-exposure to TBZ (Fig. 2B).

To further address the microtubule-associated functions of Cgp1, we examined its cellular localization. To this end, we constructed the *cgp1*Δ::*CGP1*-*mCherry* strain, where the *CGP1* gene was fused in-frame with the *mCherry* gene and introduced into the *cgp1*Δ mutant. As the *cgp1*Δ::*CGP1*-*mCherry* strain had wild type levels of TBZ susceptibility, we concluded that the Cgp1-mCherry protein is functional (Fig. S4). To visualize the microtubules, *C. neoformans* cells were stained with Tubulin Tracker™ Green. Under basal conditions, microtubules appeared to be filamentous within the cytoplasm. In response to TBZ, the filamentous microtubules appeared to be destabilized and formed punctate structures (Fig. 2C). Under both basal and TBZ-treated conditions, Cgp1 appeared to be co-localized with microtubules (Fig. 2C), indicating that Cgp1 is likely to interact with both microtubules and monomeric tubulin (Fig. 2C).

To further address the role of Cgp1 in microtubule stability, we deleted *CGP1* in the GFP-tagged β-tubulin (GFP-tubulin) strain (Fig. S5). As expected, the GFP-tubulin strain exhibited some filamentous microtubules under basal conditions, but did not exhibit such filamentous microtubules in response to TBZ and benomyl (Fig. 2D). In contrast, deletion of *CGP1* abolished filamentous microtubule formation even under basal conditions (Fig. 2D). In fact, the GFP signal was very weak in the *cgp1*Δ GFP-tubulin strain (Fig. 2D). Taken together, these results indicate that Cgp1 is a microtubule-associated protein involved in microtubule stability in *C. neoformans*.

### Cgp1 plays roles in proper nuclear segregation during mitosis and genotoxic stress responses

Eukaryotic cells use mitotic spindles, which are largely composed of microtubules, to segregate their chromosomes during cell division. Therefore, abnormal microtubule-associated functions by deletion of *CGP1* may hamper this cell cycle process in *C. neoformans*. To address this possibility, we examined the morphology of the wild type and *cgp1*Δ mutant cells upon TBZ or benomyl treatment. When we checked the percentage of cells arrested at metaphase (the mother cell still attached to the daughter cell like a dumbbell shape), we did not find any significant morphological differences between the wild type and the *cgp1*Δ mutant cells (data not shown). However, when we checked the percentage of multinucleate cells, the *cgp1*Δ mutant had much higher percentage of multinucleate cells than the wild type cells (Table 1). Treatment of wild type cells with TBZ or benomyl also moderately increased the percentage of multinucleate cells, albeit to a lesser extent than the deletion of *CGP1* (Fig. 2D and Table 1). These results indicate that the destabilization of microtubules by deletion of *CGP1* increases the number of cells with abnormal nuclear positioning and division.

**Table 1.** Percentage of cells showing each number of nuclei.

% of cells				
Strain	Condition	N	2N	3N
WT	Basal	97	3	0
WT	Benomyl treatment for 90 min	90	8	2
WT	TBZ treatment for 90 min	92	6	2
*cgp1*Δ	Basal	74	22	4
*cgp1*Δ	Benomyl treatment for 90 min	84	11	5
*cgp1*Δ	TBZ treatment for 90 min	78	21	1

**Figure 3.**
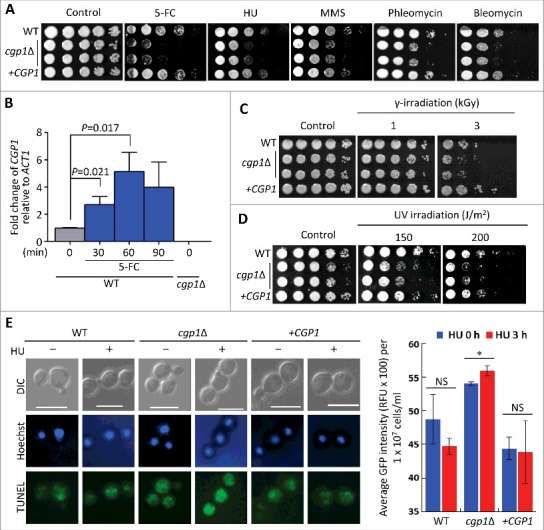
Cgp1 contributes to genotoxic stress response and adaptation. (A) Each strain [WT (H99), *cgp1*Δ mutants (YSB1631 and YSB1632), and the *cgp1*Δ::*CGP1* complemented strain (+*CGP1*, YSB3332)] was grown overnight in liquid YPD medium. Next, cells were serially diluted 10-fold, and spotted onto YPD agar medium containing the indicated concentration of flucytosine (5-FC, 500 μg/mL), hydroxyurea (HU, 100 mM), methyl methanesulfonate (MMS, 0.04%), phleomycin (0.3 μg/mL), or bleomycin (0.2 μg/mL). Cells were further incubated at 30°C and photographed daily for 2–4 days. (B) Quantitative RT-PCR examining the expression of *CGP1* in response to 5-FC treatment. *C. neoformans* wild type strains (H99) grown to the mid-logarithmic phase were treated with 25 µg/mL 5-FC, incubated for 90 min, and a portion of the cells was removed for total RNA isolation and qRT-PCR analysis. Three-independent experiments were performed. The *cgp1*Δ mutant strain (YSB1631) was used as a negative control strain. Actin was used to normalize the expression of *CGP1*. The error bar indicates SEM. The *P* value was calculated by Student's *t*-test after normalizing the data to the basal expression level. (C and D) Strains grown as described in (A) were serially diluted 10-fold, spotted (3 μL) onto YPD agar medium, exposed to the indicated dose of γ-irradiation or UV light, further cultured at 30°C, and photographed daily for 1–3 days. (E) DNA fragmentation in response to HU was labelled by TUNEL assay. Each strain [WT (H99), *cgp1*Δ mutant (YSB1632), and *cgp1*Δ::*CGP1* complemented strain (+*CGP1*, YSB3332)] was grown to mid-logarithmic phase at 30°C in liquid YPD medium and treated with (+) or without (–) 100 mM hydroxyurea for 3 h. Cells of each strains were labeled with Hoechst (blue fluorescence) and TUNEL (green fluorescence), and analyzed by fluorescence microscopy. Merged picture (Hoechst and TUNEL) analyzed using NIS-Elements software ver. 4 (Nikon). Bars, 10 µm. The mean GFP intensity of each strain was measured by fluorescence plate reader (Victor X5) from three biological replicates. Asterisk indicate the statistical significance of differences in GFP intensity between the basal and hydroxyurea treated, calculated by Student's *t*-test. Error bars represent SEM. *, *P* < 0.05; NS, not significant (*P* > 0.05).

As abnormal microtubule-associated functions may perturb chromosome stability and genotoxic stress responses, we monitored the susceptibility of the *cgp1*Δ mutant to a variety of genotoxic agents. First, the *cgp1*Δ mutants exhibited increased susceptibility to flucytosine (5-FC), which inhibits DNA and RNA synthesis (Fig. 3A). Supporting this finding, *CGP1* expression was induced by 5-FC treatment (Fig. 3B). The *cgp1*Δ mutants also exhibited increased susceptibility to other DNA damaging agents, including hydroxyurea (HU; a ribonucleotide reductase inhibitor blocking DNA synthesis [24]), methyl methanesulfonate (MMS; a DNA-alkylating agent which causes DNA fragmentation by inducing DNA double strand breaks [25]), phleomycin, and bleomycin (DNA breakage inducer [26,27]), (Fig. 3A), further corroborating the role of Cgp1 in genotoxic stress response and adaptation. Besides these DNA damaging chemical agents, the *cgp1*Δ mutants also displayed increased susceptibility to physical DNA damage caused by ionizing γ-irradiation and non-ionizing UV-irradiation. As the intensity of γ-irradiation increased (1 to 3 kGy), the *cgp1*Δ mutant had a lower survival rate than the wild type or its complemented strain (Fig. 3C). Similarly, the *cgp1*Δ mutants exhibited increased susceptibility to UV-irradiation (Fig. 3D). To examine whether the deletion of *CGP1* facilitates apoptosis-like cell death by genotoxic stress-induced DNA damage, the wild type and *cgp1*Δ mutant strains were treated with HU and then analyzed using a TUNEL (TdT-mediated dUTP nick end labeling) assay, which measures levels of DNA fragmentation caused by apoptotic cell death. The *cgp1*Δ mutant exhibited much higher levels of DNA fragmentation in response to HU than the wild type or its complemented strains (Fig. 3E). Taken together, Cgp1 is required for proper nuclear positioning and the response/adaptation to chemical and physical genotoxic stresses in *C. neoformans*.

### Cgp1 is required for maintenance of membrane stability in *C. neoformans*

**Figure 4.**
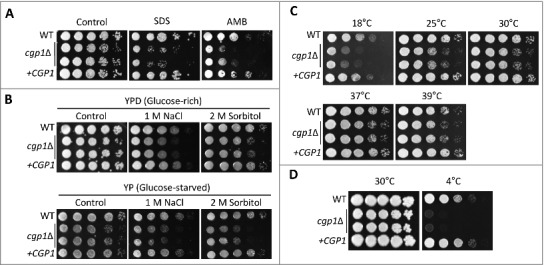
Cgp1 promotes cell membrane integrity. Each *C. neoformans* strain [WT (H99), *cgp1*Δ mutants (YSB1631 and YSB1632), and the *cgp1*Δ::*CGP1* complemented strain (+*CGP1*, YSB3332)] was grown overnight in liquid YPD medium at 30^o^C. Strains were serially diluted 10-fold, and spotted (3 μL) onto YPD agar containing the indicated concentrations of (A) SDS (0.03%) or amphotericin B (AMB, 1 µg/mL), (B) YPD (glucose-rich) or YP (glucose-starved) agar containing the indicated concentration of NaCl and sorbitol or (C and D) YPD at various temperatures. Cells were further incubated at 30°C for 2–4 days and photographed daily.

Microtubules play a role in membrane organization and trafficking pathways [1]. Microtubule-based vesicle transport driven by dynein and kinesin regulates the formation and dynamics of the cell membrane [28]. To address the role of Cgp1 in the maintenance of membrane stability, we monitored the susceptibility of the *cgp1*Δ mutants to sodium dodecyl sulfate (SDS), a membrane-destabilizing agent, and amphotericin B (AMB), which disrupts membrane stability by binding the membrane-bound sterol ergosterol [29]. The *cgp1*Δ mutants exhibited increased susceptibility to SDS and AMB compared to the wild type and its complemented strain (Fig. 4A). As membrane stability is affected by osmotic stress, we further examined the survival of the *cgp1*Δ mutants under osmotic stress conditions. The *cgp1*Δ mutants exhibited decreased growth in YPD (glucose-rich) or YP (glucose-starved) medium in the presence of different concentrations of osmotic stress agents (Fig. 4B). In particular, the fact that the *cgp1*Δ mutants exhibited increased susceptibility to 2 M sorbitol indicates that Cgp1 is likely to be involved in adaptation to osmotic stress *per se*, rather than salt stress (1 M NaCl).

Maintenance of membrane stability is critical for survival of *C. neoformans* at different temperature. When we monitored growth of *cgp1*Δ mutants in cold and high temperatures (18°C to 39°C), the *cgp1*Δ mutants exhibited much more reduced growth at low temperature (18°C and 25°C) than high temperature (Fig. 4C). This cold temperature sensitivity of the *cgp1*Δ mutant was more evident at 4°C. The wild type and *cgp1*Δ*::CGP1* complemented strains grew slowly at 4°C, but the *cgp1*Δ mutant failed to grow (Fig. 4D). Taken together, these data support the notion that Cgp1 is involved in the maintenance of membrane stability.

### Cgp1 is involved in virulence of *C. neoformans*

**Figure 5.**
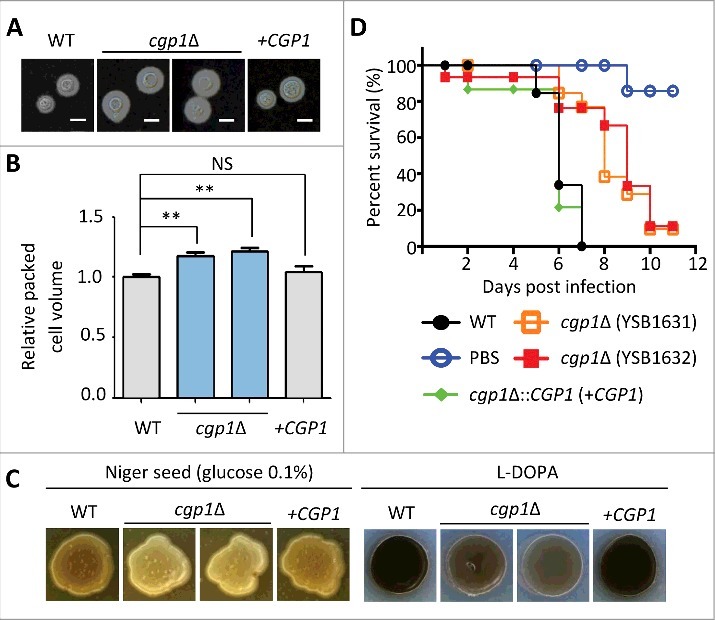
Cgp1 is required for *C. neoformans* virulence (A) For qualitative measurement of capsule production, each *C. neoformans* strain [WT (H99), *cgp1*Δ mutants (YSB1631 and YSB1632), and the *cgp1*Δ::*CGP1* complemented strain (+*CGP1*, YSB3332)] was grown overnight and then spotted onto solid DME agar medium and further incubated at 37°C for 2 days. Cells were fixed, stained by India ink, and observed by microscope. The scale bars represent 10 μm. (B) For quantitative measurement of capsule production, cells were loaded into capillary tubes, which were subsequently placed vertically at room temperature. The relative packed capsule volume was measured by calculating the ratio of the length of packed cell volume phase to the length of the total volume phase. Three independent experiments with technical triplicates were performed. Asterisk indicate the statistical significance of difference in relative packed cell volume (**, *P* < 0.01; NS, not significant). The *P* value was calculated by one-way analysis of variance with Bonferroni's multiple-comparison test. (C) *C. neoformans* strains [WT (H99), *cgp1*Δ mutants (YSB1631 and YSB1632), and the *cgp1*Δ::*CGP1* complemented strain (+*CGP1*, YSB3332)] were cultured in liquid YPD medium at 30^o^C for 16 h. Cells were then spotted onto the solid Niger seed medium (left panel) containing the indicated concentration of glucose. Cells were also spotted onto the L-DOPA medium (right panel) and further incubated at 25^o^C. Photographs were taken at 2 days. (D) Insect-based virulence test. The four *C. neoformans* strains described in (A) and PBS (non-infection control) were injected into *Galleria mellonella* (15 larvae per group) and survival was determined. The *P* values calculated using the Log-rank test between strains were as follows: *P* = 0.0010 for WT vs. *cgp1*Δ mutant (YSB1631), *P* < 0.0001 for WT vs. *cgp1*Δ mutant (YSB1632), and *P* = 0.5645 for WT vs. *cgp1*Δ::*CGP1* (YSB3332).

Next, we addressed the role of Cgp1 in virulence factor production and the *in vivo* virulence of *C. neoformans. C. neoformans* has two major virulence factors, melanin and capsule. The capsule prevents cryptococcal cells from being phagocytized by host innate immune cells [30]. Melanin pigment not only serves as an antioxidant, but also confers antiphagocytic activity [31]. Although a striking difference in capsule production was not observed between wild type and *cgp1*Δ mutant strains (Fig. 5A), careful quantitative measurement of capsule production revealed that the *cgp1*Δ mutants had a slightly increased level of capsule production compared to the wild type and the complemented strain (Fig. 5B and Fig. S6), suggesting that Cgp1 may negatively affect capsule production, or that Cgp1 may also alter the composition or structure of the capsule. In contrast to capsule production, *cgp1*Δ mutants exhibited reduced melanin production in both Niger seed and L-DOPA media (Fig. 5C).

To examine the role of Cgp1 in *C. neoformans* virulence, we employed an insect killing assay using the wax moth *Galleria mellonella* [32,33]. The group of wax moths infected with the *cgp1*Δ mutants had a higher survival rate than those infected with the wild type or the complemented strain (Fig. 5D). Taken together, Cgp1 is required for the production of virulence factors melanin and capsule and virulence in a moth model.

### Cgp1 plays dual roles in sexual differentiation of *C. neoformans*

**Figure 6.**
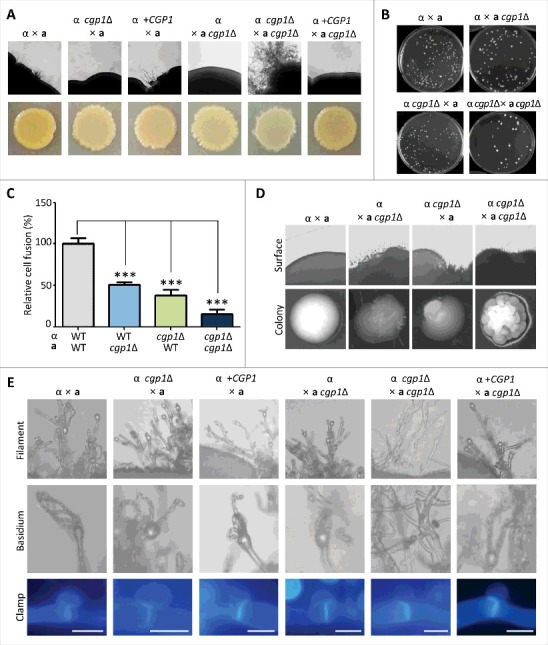
Cgp1 regulates sexual differentiation in both a positive and a negative manner at different developmental stages in *C. neoformans*. (A) Cgp1 is negatively involved in filament formation. The WT *MATα* and *MAT***a** type strains (H99 and KN99**a**), the *MATα* *cgp1*Δ mutant (YSB1632), the *MATα* *cgp1*Δ::*CGP1* complemented strain (+*CGP1*, YSB3332) and the *MAT***a** *cgp1*Δ mutant (YSB3889), were co-cultured on V8 agar medium at room temperature in the dark for 10 days and photographed daily. (B and C) Cell fusion assay. Cells of opposite mating types were spotted onto V8 agar medium, incubated for 24 hr at room temperature in the dark, scraped, resuspended in PBS, and spread onto YPD plate containing both nourseothricin and G418. The plates were incubated for 3–4 days at 30°C and the colonies were counted. (C) Cell-fusion efficiency of unilateral and bilateral crossing was calculated relative to that of control strains [*NAT*-marked WT*α* strain (YSB119) x *NEO*-marked WT**a** strain (YSB121)]. Three independent experiments with technical triplicates were performed. (***: *P* value < 0.001, the error bar indicates SEM). The *P* value was calculated by one-way analysis of variance with Bonferroni's multiple-comparison test. (D) Morphology of dikaryotic colonies after cell fusion. Colonies grown on a YPD plate containing both nourseothricin and G418 (B) were observed under light microscopy (Olympus BX51 microscope). (E) Microscopic examination of mating structure produced in *cgp1*Δ mutants and wild type during mating process. Cells mixed with each mating partner were streaked in a straight line on V8 agar medium. Filamentous growth was observed at 6 day after mating, and basidium, basidiospores, or clamp connection was observed at 9 day after mating. The clamp connection was stained with 2% calcofluor-white during 30 min for fluorescent visualizing.

*C. neoformans* infection can be initiated by the inhalation of infectious basidiospores generated through bisexual differentiation between cells of opposite mating types [34–36]. During this developmental process, dynamic regulation of the cytoskeleton is likely to occur [37,38]. To examine the role of Cgp1 in the developmental process, we constructed the *cgp1*Δ mutant in the *MAT***a** serotype A *C. neoformans* var. *grubii* KN99**a** strain (Fig. S2). Notably, a bilateral cross between *MAT***a** *cgp1*Δ and *MAT*α *cgp1*Δ mutants allowed cells to produce more prolific and longer filaments compared to those seen in wild type strains (Fig. 6A). This finding was rather surprising and unexpected because the *bim1*Δ mutant exhibits reduced filamentous growth [39]. This finding prompted us to address whether Cgp1 affects the cell-cell fusion process. Strains differentially marked with distinct selection markers (*MAT***a** with *NEO* and *MATα* with *NAT*) were crossed and the number of dikaryonic colonies that are able to grow on medium containing nourseothricin and G418 were compared. In stark contrast to the negative role of Cgp1 in filamentous growth, a bilateral cross between *MAT***a** *cgp1*Δ and *MATα* *cgp1*Δ mutants resulted in severe defects in cell fusion compared to a similar cross between wild type strains (Fig. 6B and C). A unilateral cross between *cgp1*Δ and wild type strains revealed intermediate efficiency in cell fusion. Interestingly, dikaryonic colonies produced by a bilateral cross between *cgp1*Δ mutants exhibited highly enhanced filamentation at the periphery of the colonies (Fig. 6D), suggesting that the *cgp1*Δ mutants were highly enhanced in filamentous growth once they undergo cell fusion.

Next, we addressed whether deletion of *CGP1* affects clamp connection and formation of basidia and basidiospores after filamentation. In wild type or unilateral crosses, normal clamp connection at the septum and basidium formation with four chains of basidiospores at the tip of filament were observed (Fig. 6E). In the bilateral cross between *cgp1*Δ mutants, however, neither of basidia nor basidiospores were observed in spite of the presence of normal clamp connection (Fig. 6E), indicating that Cgp1 is required for basidia and basidiospore formation. Taken together, these data suggest that Cgp1 plays dual roles in sexual differentiation: positive roles in cell fusion and basidia/basidiospore formation and negative roles in filamentous growth.

### The CAP-Gly domain is required for all cellular functions of Cgp1

**Figure 7.**
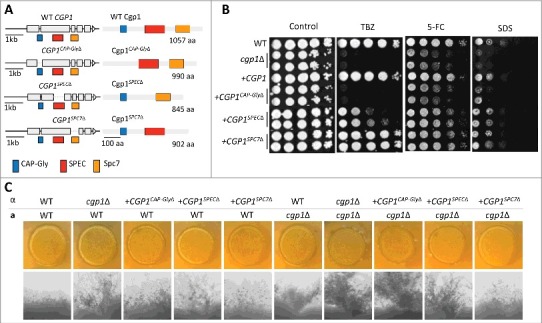
The CAP-Gly domain is required for the function of Cgp1. (A) Schematic diagram of domain deletion construction (*cgp1*Δ::*CGP1^CAP-Gly^*^Δ^, *cgp1*Δ::*CGP1^SPEC^*^Δ^, *cgp1*Δ::*CGP1^SPC7^*^Δ^). (B) The role of each Cgp1 domain in microtubule stability and genotoxic stress responses. Each strain [WT (H99), *cgp1*Δ mutants (YSB1631 and YSB1632), the *cgp1*Δ::*CGP1* complemented strain (+*CGP1*, YSB3332), *cgp1*Δ::*CGP1^CAP-Gly^*^Δ^ strains (+*CGP1^CAP-Gly^*^Δ^, YSB3897 and YSB3901), *cgp1*Δ::*CGP1^SPEC^*^Δ^ strains (+*CGP1^SPEC^*^Δ^, YSB3949 and YSB3957), and *cgp1*Δ::*CGP1^SPC7^*^Δ^ strains (+*CGP1^SPC7^*^Δ^, YSB3905 and YSB3907] was grown on YPD medium. The grown cells were serially diluted 10-fold and spotted onto YPD agar medium containing the indicated concentration of TBZ (7.5 µg/mL), 5-FC (300 µg/mL) or SDS (0.03%), further incubated at 30°C and photographed daily for 2–4 days. (C) The function of each Cgp1 domain in *C. neoformans* sexual differentiation. The following *MAT*α and *MAT***a** strains were crossed unilaterally or bilaterally on V8 agar medium and cultured at room temperature in the dark for 10 days and photographed daily: WT*α* (H99), WT**a** (KN99**a**), *α* *cgp1*Δ (YSB1632), **a** *cgp1*Δ (YSB3889), *α* *cgp1*Δ::*CGP1^CAP-Gly^*^Δ^ (YSB3897), *α* *cgp1*Δ::*CGP1^SPEC^*^Δ^ (YSB3949), and *α* *cgp1*Δ::*CGP1^SPC7^*^Δ^ (YSB3905).

As Cgp1 has pleiotropic roles in *C. neoformans*, we questioned which domain of Cgp1 is responsible for these functions. Cgp1 contains three separate protein domains: CAP-Gly, SPEC, and Spc7 (Fig. 1A). To dissect the function of each domain, we constructed domain deletion strains, in which each domain was selectively deleted (Fig. 7A). Each domain deletion construct was introduced into the *cgp1*Δ strain by targeting to the native locus of *CGP1* (*cgp1*Δ::*CGP1^CAP-Gly^*^Δ^, *cgp1*Δ::*CGP1^SPEC^*^Δ^ and *cgp1*Δ::*CGP1^SPC7^*^Δ^), in the same manner used to construct the complemented strains. We confirmed the correct expression of each truncated *CGP1* by qRT-PCR (Fig. S7). Under all tested conditions, the *cgp1*Δ::*CGP1^CAP-Gly^*^Δ^ strain displayed phenotypes similar to those of the *cgp1*Δ mutant strain (Fig. 7B, C and Fig. S8), suggesting that the CAP-Gly domain is essential for all the functions of Cgp1 that we assessed. In contrast, the phenotypes of the *cgp1*Δ::*CGP1^SPEC^*^Δ^ and *cgp1*Δ::*CGP1^SPC7^*^Δ^ strains were generally similar to those of wild type strain, with the exception of TBZ susceptibility and filamentous growth. The *cgp1*Δ::*CGG1^SPEC^*^Δ^ strain, but not the *cgp1*Δ::*CGP1^SPC7^*^Δ^ strain, only partially restored normal TBZ resistance (Fig. 7B), suggesting that the SPEC domain plays a minor role in microtubule stabilization. Furthermore, the *cgp1*Δ::*CGG1^SPEC^*^Δ^ strain displayed weakly enhanced filamentous growth (Fig. 7C). The SPEC domain may therefore cooperate with the CAP-Gly domain to regulate some functions of Cgp1. Overall, these data demonstrated that the CAP-Gly domain is critical for Cgp1 function, the SPEC domain plays a minor role, and the Spc7 domain appears dispensable for Cgp1 functions.

### Overexpression of *CGP1* renders *C. neoformans* sensitive to diverse environmental stresses

**Figure 8.**
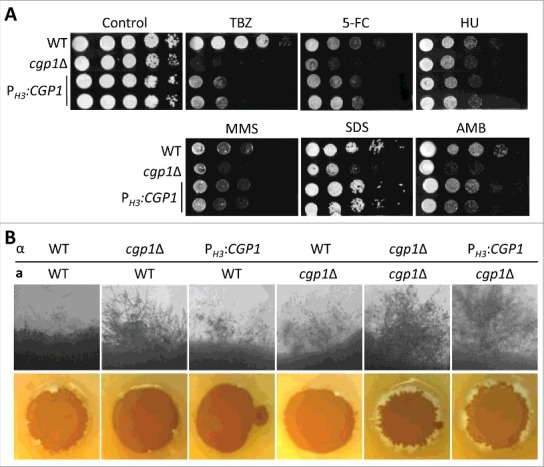
Overexpression of *CGP1* perturbs microtubule stability and normal mating efficiency. (A) *CGP1* overexpression decreases microtubule stability. The WT (H99), the *cgp1*Δ mutant (YSB1631), and the *CGP1* overexpression strains (P*_H3_*:*CGP1*, YSB3663 and YSB3665) were cultured overnight in YPD medium, serially diluted 10-fold, and spotted onto YPD agar medium containing the indicated concentration of TBZ (7.5 µg/mL), 5-FC (500 µg/mL), HU (90 mM), MMS (0.03%), SDS (0.03%), or AMB (1 µg/mL). (B) *CGP1* overexpression promotes filament formation. The following *MATα* and *MAT***a** strains were crossed unilaterally or bilaterally on V8 agar medium and cultured at room temperature in the dark for 10 days and photographed daily: WT*α* (H99), WT**a** (KN99**a**), *α* *cgp1*Δ (YSB1632), **a** *cgp1*Δ (YSB3889), *α* P*_H3_*:*CGP1* (YSB3663).

To address whether increased expression of *CGP1* enhances the microtubule-related functions described thus far, we constructed *CGP1* overexpression strains (P*_H3_*:*CGP1*) by replacing the native *CGP1* promoter with the histone 3 promoter (Fig. S9). We confirmed that *CGP1* was more than 10-fold overexpressed in the P*_H3_*:*CGP1* strains than the wild type strain (Fig. S9). Overexpression of *CGP1* did not affect wild type levels of resistance to other DNA damaging agents (5-FC, HU, or MMS) and membrane destabilizers (SDS or AMB) (Fig. 8A). However, P*_H3_*:*CGP1* strains displayed increased susceptibility to TBZ, albeit not to the extent of the *cgp1*Δ mutant (Fig. 8A). Furthermore, overexpression of *CGP1* also enhanced filamentous growth in *C. neoformans*, as was noted for deletion of *CGP1* (Fig. 8B). These findings suggest that balanced expression of Cgp1 is required to maintain normal microtubule stability and mating efficiency.

### Elucidation of Cgp1-dependent regulatory networks

Given the pleiotropic roles of Cgp1 in *C. neoformans*, we attempted to elucidate Cgp1-dependent regulatory networks by identifying Cgp1-interacting proteins. To this end, we constructed the *cgp1*Δ::*CGP1*-*4xFLAG* strain to perform an *in vitro* pull down assay. The *cgp1*Δ::*CGP1*-*4xFLAG* strain was phenotypically identical to the *cgp1*Δ::*CGP1* complemented strain, suggesting that the Cgp1-4xFLAG protein is functional (Fig. S4).

**Figure 9.**
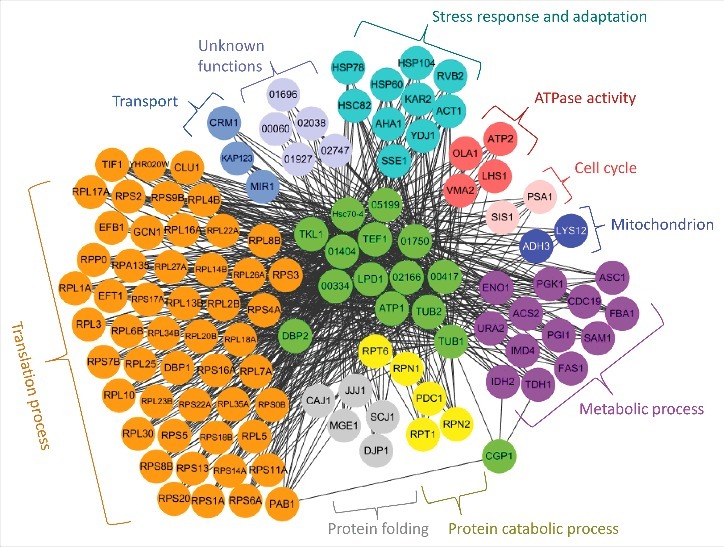
Cgp1-related regulatory networks. Functional network analysis of the Cgp1-related proteins in *C. neoformans* using CryptoNet. This gene-function correlation was based on 100 function-related candidate genes to Cgp1-interacting proteins as a gene query, and the network picture was drawn using Cytoscape 3.2.1 version. Genes in this network were classified based on their Gene Ontology (GO) term or their predicted biological functions listed in Table S4.

*In vitro* pull-down of the Cgp1-4xFLAG protein with anti-FLAG antibodies followed by proteomics analysis revealed several Cgp1-interacting proteins (Fig. S10 and Table S3). In agreement with co-localization data between Cgp1 and microtubules (Fig. 2C), tubulins (α-tubulin encoded by CNAG_03787 and β-tubulin encoded by CNAG_01840) were included in the list of Cgp1-interacting proteins (Table S3), which further verified the direct interaction between microtubules and Cgp1. To further elucidate the Cgp1-related functional networks, we used a genome-scale co-functional network in *C. neoformans*, CryptoNet (www.inetbio.org/cryptonet) [40], using the identified Cgp1-interacting protein candidates as queries (Table S4). Any proteins that were identified to be functionally linked to Cgp1 and its interacting proteins by CryptoNet were categorized by Gene Ontology (GO) terms and also by previously published information. As a result, we obtained a comprehensive Cgp1-related regulatory network (Fig. 9). The biological functions covered by this network include translation process, protein folding/catabolic process, stress response and adaptation, transport, cell cycle, ATPase activity, and other metabolic process. These data further support the diverse biological roles of Cgp1 in *C. neoformans*.

## Discussion

The CAP-Gly domain is present in a number of eukaryotic microtubules-associated proteins and functions by interacting with tubulin monomers, tubulin dimers, and/or microtubules to regulate microtubule organization and dynamics. In addition, CAP-Gly proteins are involved in intracellular signaling, distribution of membrane organelles, and cell architecture [6]. Nevertheless, the functions of CAP-Gly proteins have not been well studied in fungal pathogens. In this study, we identified five CAP-Gly proteins in *C. neoformans* namely, Cgp1, Alf1, Nip100, Pac2, and Bsp3. Among these, we selected Cgp1 for in-depth functional analyses due to its unique domain structure.

This study demonstrated that Cgp1 (CNAG_06352) was critical for microtubule maintenance and other biologically important functions in *C. neoformans*, such as growth, stress responses, virulence factor production, and differentiation. These pleiotropic Cgp1 roles may explain why the *cgp1*Δ mutant had attenuated virulence. During preparation of our manuscript, Maier *et al.* reported a partial functional analysis of CNAG_06352 and classified it as a transcription factor, which they termed Bik1 based on the homology to *S. cerevisiae* Bik1 [41]. Brown *et al.* have also reported the chemical susceptibility of the CNAG_06352 deletion mutant, and described the presence of bZIP, CAP-Gly and Pox A type domains in CNAG_06352 [42]. However, the data presented in this report demonstrate that CNAG_06352 is not likely to be a transcription factor. First, in our Pfam analysis, CNAG_06352 does not have any apparent DNA binding domain. In support of this, the current annotation for CNAG_06352 in the *C. neoformans* genome database is ‘hypothetical protein’. Second, the top Blast hit for CNAG_06352 in the *S. cerevisiae* genome database (SGD, http://www.yeastgenome.org/) is not Bik1 (score 47.3; e-value 2.9E^−13^), but Uso1 (score 87.4, e-value 6.2E^−17^), although the closest Bik1 ortholog in *C. neoformans* is CNAG_06352. Furthermore, Bik1 and Uso1 are not transcription factors in *S. cerevisiae*. Third, although Bik1 has similar microtubule-related functions in *S. cerevisiae*, its structural features are different from CNAG_06352. The protein size of Bik1 is 440 amino acids (∼51 KDa) whereas CNAG_06352 is 1,058 amino acids (∼114 KDa). Bik1 contains a single CAP-Gly domain at the N-terminus but does not have SPEC and Spc7 domains as CNAG_06352 does. Fourth, our study demonstrates that CNAG_06352 is mainly co-localized to the microtubule, not to the nucleus, and is involved in microtubule stability. Accordingly, here we have re-named CNAG_06352 as Cgp1 (CAP-Gly protein 1), not Bik1.

Here we found that Cgp1 is required for proper nuclear segregation during mitosis. Deletion of *CGP1* did not significantly affect cell morphology, but increased the number of multinucleate cells, probably due to destabilized microtubules and/or interrupted spindle assembly checkpoint (SAC). This is rather unexpected finding, because in other eukaryotes, microtubule depolymerizing agents such as TBZ and benomyl arrest cells at metaphase and cause significant morphological change [23,43–45]. In the budding yeast *S. cerevisiae*, nuclear microtubules are required for spindle assembly, chromosome segregation, and the cytoplasmic microtubules are required for proper nuclear positioning and division [46,47]. Although distantly related to *C. neoformans* Cgp1, *S. cerevisiae* Bik1 is also required for proper spindle formation and nuclear segregation and thereby deletion of *BIK1* generates many multinucleate cells [11], suggesting some conserved roles of Cgp1 and Bik1 in the two ascomycete and basidiomycete budding yeasts. The potential role of Cgp1 in the SAC response will be examined in future studies.

We believe that the function of Cgp1 in the genotoxic stress response and adaptation could also be attributed to the proper formation of microtubular spindles during cell division. The mitotic spindle composed of microtubules is required for equal separation of the chromosomes in a parental cell into two daughter cells during cell division. During the cell cycle, there are two checkpoints, which are spindle and DNA damage checkpoints, that cooperate to delay mitosis and preserve genome integrity [48]. The defective spindle stability caused by deletion of *CGP1* may influence the spindle checkpoints, which may subsequently affect the DNA damage checkpoints, and further result in a failure of DNA repair. The perturbation of proper DNA repair in response to genotoxic stresses may cause apoptotic cell death in *C. neoformans*, as shown here that the *cgp1*Δ mutant exhibited much higher levels of apoptotic cell death than the wild type strains. Similarly, it has been reported that hydrogen peroxide, which is able to confer DNA damage as reactive oxygen species, induces apoptosis-like cell death in both serotype A and D *C. neoformans* [49,50].

Interestingly, the key functions of Cgp1 appear to be mainly contributed by the CAP-Gly domain even though Cgp1 has three distinct protein domains. To support this, the phenotypes of *cgp1*∆ mutants were similar to those of the *cgp1*Δ::*CGP1^CAP-Gly^*^Δ^ strains. All the Cgp1 orthologs in the pathogenic *Cryptococcus* species complex have an Spc7 domain. The function of the Spc7 protein, but not the Spc7 domain itself, has been studied in the fission yeast *S. pombe*. In the fission yeast, Spc7 interacts with the EB1 homologue Mal3 and is required for microtubule-kinetochore interaction [51,52]. However, our present study revealed that deletion of the Spc7 domain did not affect microtubule-associated function and sexual differentiation. Based on this evidence, we can speculate that Cgp1 has the Spc7 domain, which has been evolutionarily derived from Spc7, but has lost its function during evolution. The SPEC domain is a spectrin repeat that is found in several proteins involved in cytoskeletal structure [53]. From our functional domain analysis, the SPEC domain in Cgp1 plays only a minor role in microtubule stability and filamentous growth. According to the work of Djinovic-Carugo *et al*., an interaction exists between the microtubule-associated protein Tau and spectrin [54]. Therefore, it is possible that the SPEC domain may play an additional role in regulating the microtubule-associated functions in Cgp1, just as the CAP-Gly domain does. Further studies need to be conducted to explore this possibility.

Previously the function of another microtubule-binding protein, Bim1, has been reported in *C. neoformans* by Staudt *et al.* [39]. *C. neoformans* Bim1 is orthologous to *S. cerevisiae* Bim1 and contains a Calponin Homology (CH) domain for actin binding and an EB1-like domain for microtubule binding. Similar to Cgp1, deletion of *BIM1* increases susceptibility to a microtubule destabilizer (benomyl), suggesting its critical role in microtubule stability. However, the *cgp1*Δ mutant is phenotypically different from the *bim1*Δ mutant, which suggests that phenotypes observed in these mutants may not simply result from abnormal microtubule regulation. For example, deletion of *BIM1* does not cause any growth defects at different growth temperatures [39], whereas deletion of *CGP1* affects normal cell growth, particularly at low temperatures. Most notably, Bim1 only plays a positive role in sexual differentiation in *C. neoformans* [39], whereas Cgp1 plays both positive and negative roles at different developmental stages of the pathogen. In this regard, Bim1 mainly acts after cell fusion and promotes the formation of filaments [39]. Therefore, deletion of *BIM1* leads to poorer and shorter filament formation, and premature entry into the later sexual stages. In contrast, Cgp1 directly promotes cell fusion at the early developmental stage while also suppressing filamentous growth at the later stages of sexual differentiation. After filamentation, however, Cgp1 is also required for basidia and basidiospore formation. These results suggest that diverse microtubule-binding proteins play distinct roles at different developmental stages in *C. neoformans*.

In this study, we demonstrated that the CAP-Gly domain is absolutely required for all the functions of Cgp1. In fact, the CAP-Gly domain is known not only to bind to the EEY/F-COO- sequence motifs in α-tubulin, but also to interact with other domains, including end-binding homology domains, zinc-finger motifs and proline-rich domains [6], suggesting that CAP-Gly proteins could be involved in multiple biological processes as well as microtubule-related functions. Supporting this, we also discovered a number of other Cgp1-interacting proteins, in addition to expected α-tubulin, through an *in vitro* pull-down analysis using FLAG-tagged Cgp1. The CryptoNet analysis revealed that biological functions covered by Cgp1-related networks included translation, protein folding/catabolism, transport, stress response and adaptation, cell cycle, ATPase activity, mitochondrion function, and other metabolic processes. In future studies, the pathobiological functions of Cgp1-interacting proteins should be further elucidated.

Given the pleiotropic roles of Cgp1 in *C. neoformans*, it was not surprising that the *cgp1*Δ mutant had attenuated virulence. In particular, defects in melanin synthesis, growth at high temperature, and general stress responses, may collectively contribute to the attenuated virulence of the *cgp1*Δ mutant. Based on its structural difference compared to other evolutionarily conserved CAP-Gly proteins, Cgp1 or its interacting proteins could be further exploited as novel antifungal drug targets. In conclusion, our study shows that a unique microtubule-associated CAP-Gly protein, Cgp1, plays pivotal roles in the pathobiological functions of the global human meningitis fungal pathogen *C. neoformans*.

## Materials and methods

### Construction of *cgp1*Δ mutant and its complemented strains

The *CGP1* gene was disrupted in the *C. neoformans* serotype A *MATα* strain (H99), the *MAT***a** strain (KN99**a**) or LK126 (GFP-tubulin strain in the H99 background) using double-joint PCR (DJ-PCR) with *NAT* or *NEO*-split markers and biolistic transformation [55]. The scheme illustrating the *CGP1* gene disruption is shown in Fig. S2, Fig. S5 and the primers used are listed in Table S2. The dominant selectable markers (*NAT* or *NEO*) were amplified with primer M13Fe (M13 forward extended) and M13Re (M13 reverse extended) from plasmid pNAT or pJAF1, respectively. In the first round of PCR, the 5- or 3’- flanking regions of *CGP1* were amplified using primer pairs B3714/B3715 and B3716/B3717, respectively, with H99 or KN99**a** genomic DNA as a template respectively. In the second round of PCR, the *CGP1* disruption cassettes with 5’- or 3’- regions of the *NAT* or *NEO* split markers were amplified by DJ-PCR with the primer pairs B3714/B1455, B3717/B1454, B3714/B1887, and B3717/B1886, using the first-round PCR product as a template. The 5’- and 3’- split deletion cassettes were precipitated onto 0.6 µm gold microcarrier beads (Bio-Rad) and introduced into the H99, KN99**a** or LK126 strains by biolistic transformation [56]. Stable transformants were selected on YPD medium containing nourseothricin or G418 and were initially screened by diagnostic PCR with the screening primers listed in Table S2 and positive mutant strains were further confirmed using Southern blot analysis with genomic DNA digested with the appropriate restriction enzymes as shown in Fig. S2 and Fig. S5.

To verify the phenotypes observed in the *cgp1*Δ mutants, *cgp1*Δ::*CGP1* complemented strains were constructed by re-integrating the wild type *CGP1* gene into the native *CGP1* locus (Fig. S3). First, the full-length *CGP1* gene containing the promoter, the open reading frame (ORF), and the terminator were amplified using primers B6102/B6103 containing a NotI restriction site with H99 genomic DNA as the template. The PCR products were then cloned into pTOP-V2 (Enzynomics) to generate the plasmid pTOP-CGP1. After confirming the DNA sequence, the NotI-digested *CGP1* insert was sub-cloned into plasmid pJAF12 containing the *NEO* selectable marker to produce plasmid pJAF12-CGP1. Next, pJAF12-CGP1 was linearized by PmII digestion and the linearized pJAF12-CGP1 was introduced by biolistic transformation into the native *CGP1* locus of the *cgp1*Δ mutant (YSB1632). The targeted re-integration of the *CGP1* gene into its native locus was confirmed by diagnostic PCR with the primer pair B3713/B6104.

### Construction of *cgp1*Δ::*CGP1*-*GFP, cgp1*Δ::*CGP1*-*4xFLAG*, and *cgp1*Δ::*CGP1*-*mCherry*-tagged strains

The full-length *CGP1* gene without the 3′ untranslated region was amplified by PCR using genomic DNA as the templates and the primers B6102/B6121 containing a NotI restriction site. The PCR products were then cloned into the pTOP-V2 plasmid, sequenced, and subsequently transferred into pNEO-GFPht, pNEO-4×FLAG, or pHYG-mCherry plasmids [57] to generate the plasmids pNEO-CGP1-GFPht, pNEO-CGP1-4xFLAG, and pHYG-CGP1-mCherry. The plasmids were linearized by PmII digestion and biolistically transformed into the *cgp1*Δ mutant (YSB1632). To demonstrate target integration into the native *CGP1* locus of the *cgp1*Δ mutant, diagnostic PCR was performed with primers B3713/B6104. To confirm that the Cgp1-tagged constructs were biologically active, phenotypic analyses were performed (Fig. S4).

### Construction of *cgp1*Δ::*CGP1^CAP-Gly^*^Δ^, *cgp1*Δ::*CGP1^SPEC^*^Δ^, and *cgp1*Δ::*CGP1^SPC7^*^Δ^ domain deletion strains

For Cgp1 domain analysis, *CGP1* domain deletion strains were constructed as follows. To construct the *cgp1*Δ::*CGP1^CAP-Gly^*^Δ^ strain, the entire DNA sequence of the *CGP1* gene was divided into two parts for deletion of the CAP-Gly domain. A region located upstream of the CAP-Gly domain was amplified by PCR using primer B6859 containing an XmaI site and B6854 containing an NheI site. A region located downstream of the CAP-Gly domain was amplified by PCR using primer B6853 containing a NheI site and primer B6852 containing a NotI site. The PCR product derived from the upstream region was cloned into pTOP-V2 to produce the pTOP-CAP-Gly-Front plasmid, and the PCR product derived from the downstream region was cloned into pTOP-V2 to generate the pTOP-CAP-Gly-Back plasmid. Following this, the insert in plasmid pTOP-CAP-Gly-Front was sub-cloned into pJAF12 by XbaI and NotI digestion to generate plasmid pJAF12-CAP-Gly-Front. Subsequently, the insert from plasmid pTOP-CAP-Gly-Back was sub-cloned into the plasmid pJAF12-CAP-Gly-Front by NheI and NotI digestion to generate the pJAF12-CAP-Gly-Front+Back. The plasmid pJAF12-CAP-Gly-Front+Back was linearized by PmII digestion and introduced into the *cgp1*Δ mutant strain (YSB1632). The targeted re-integration of the *CGP1^CAP-Gly^*^Δ^ gene into its native locus was confirmed by diagnostic PCR. The two other domain deletion constructs were generated in a similar way using the primers listed in Table S2. The expression of the three CAP-Gly domain deletion strains was confirmed by comparison with the *cgp1*Δ mutant strain and the wild type strain by qRT-PCR (Fig. S7).

### Construction of *CGP1* overexpression strains

In order to construct the constitutive *CGP1* overexpression strains, we generated the P*_H3_*:*CGP1* cassette as follows. In the first round of PCR, the 5’-flanking and the 5’-exon regions of *CGP1* were amplified with the primer pairs B3714/B6652 and B6653/B6654, respectively. The *NAT-H3* promoter fragments were amplified with primers B4017 and B4018 using the plasmid pNAT-H3 as the template. In the second round of PCR, the left fusion fragment was amplified using primers B3714 and B1455 by combining the first round PCR products for the 5’-flanking region of *CGP1* and the *NAT-H3* promoter as templates. A right fusion fragment was amplified using primers B1454 and B6654 by combining the first round PCR products for the 5’-exon region of *CGP1* and *NAT-H3* promoter as templates. Next, the left and right fusion fragments were mixed and introduced into H99 strain by biolistic transformation (Fig. S9A). The targeted integration of the *NAT-H3* promoter insertion cassette into the native locus of *CGP1* was confirmed by diagnostic PCR and Southern blot analysis (Fig. S9B). The positive strains were further verified by measuring the expression level of *CGP1* using qRT-PCR (Fig. S9C).

### Total RNA isolation and qRT-PCR

Cells were grown in 50 mL of YPD medium at 30°C for 16 hr, sub-cultured into 200 mL of fresh YPD medium with an inoculum at optical density 600 nm (OD_600_) of 0.2, and further incubated for 4–5 hr at 30°C until the OD_600_ reached approximately 0.8. The medium was divided into 50 mL for the non-treated zero time control, and 150 mL for subsequent chemical treatment. The 150 mL of culture was treated with 25 μg/mL flucytosine (5-FC) or 7 μg/mL thiabendazole (TBZ) and further incubated at 30°C for 90 min. During the culture period, 50 mL of the culture was sampled every 30 min. Each sample was frozen and lyophilized overnight. Total RNAs were isolated using Trizol reagent as described before [58]. cDNA was synthesized from the total RNA using the MMLV reverse transcriptase (Invitrogen) and qRT-PCR was used to quantitatively measure the relative expression levels of *CGP1* using the primers listed in Table S2 using the cDNA as template. qRT-PCR was conducted using a CFX96 Real-Time PCR detection system (Bio-Rad) and *ACT1* was used for the normalization of gene expression.

### Cgp1 and tubulin localization study

For the Cgp1 localization study, the Cgp1*-*mCherry-tagged strain (YSB3964), Gfp-tubulin strain (LK126) or *cgp1*Δ GFP-tubulin strain (YSB5536) were cultured in 50 mL of liquid YPD medium overnight and sub-cultured into 200 mL of fresh YPD medium for 4 hr. After 1 mL of the culture was sampled, 7 μg/mL TBZ or 4 μg/mL benomyl was added to the remaining culture further incubated for 90 min at 30°C. After this time, 1 mL of the drug-treated culture was sampled. To visualize the Cgp1-mCherry with tubulin staining, the Cgp1*-*mCherry-tagged strain sample pellets were washed with phosphate buffered saline (PBS), resuspended in 1 mL of fresh YPD medium, and 0.5 μL of Tubulin Tracker™ Green (Thermo Fisher Scientific, Oregon Green® 488 Taxol, bis-acetate) was added and the culture was further incubated for 30 min at 37°C. The samples were stored in 2% paraformaldehyde in PBS to fix the cells. The GFP-tubulin strains were stained with Hoechst (Thermo Fisher Scientific, Hoechst 33342) for 30 min and washed with PBS. The samples were observed under a fluorescence microscope (Nikon, eclipse Ti).

### *In vitro* pull-down assay

The *cgp1*Δ::*CGP1*-4xFLAG-tagged strain (YSB3541) was cultured overnight in 50 mL of YPD medium at 30°C, inoculated into 1 L of fresh YPD medium, and further cultured for 4 hr until the OD_600_ of the culture reached approximately 0.8. Total cellular proteins were isolated using lysis buffer [50 mM Tris pH 7.5, 1% sodium deoxycholate, 5 mM sodium pyrophosphate, 0.1 mM sodium orthovanadate, 50 mM NaF, 0.1% SDS, 1% Triton X-100, 1 x proteinase cocktail inhibitor (Calbiochem, 539136), and 0.5 mM PMSF] as previously described [59]. A portion of the protein extract was retained as an input control and the remaining protein extract was incubated with an anti-FLAG antibody (Sigma-Aldrich, F1804) for 16 hr at 4°C, followed by addition of Protein G Sepharose 4 Fast Flow (GE, 17-0618-01) and further incubation at 4°C for 8 hr, after which the beads were pelleted at 4°C. A portion of the depleted supernatant was recovered as the output. The beads were washed three times with lysis buffer and heated with SDS-PAGE loading buffer at 100°C for 10 min. The beads were pelleted and the eluted proteins, along with the input and output samples, were separated by SDS gel electrophoresis. The gel was subsequently stained using Coomassie brilliant blue and stained bands were subjected to liquid chromatography-tandem mass (LC-MS/MS) analysis as previously reported [57]. Q Exactive Hybrid Quadrupole Orbitrap mass spectrometry (Thermo Fisher Scientific) was used to analyze mass spectrometry data, according to a previously described protocol [33]. MS/MS spectra were searched against the Universal Protein Resource (http://uniprot.org) using MASCOT (ver. 1.4; Thermo Fisher Scientific) for peptide assignment.

### *In vitro* virulence factor assay

Cells were incubated overnight and were then spotted (5 µL) onto DME agar medium, and further incubated for 2 days at 37°C. After incubation, the capsule was visualized with India ink staining and observed with a microscope equipped with a SPOT Insight digital camera for qualitative measurement. For quantitative measurement of the capsule, packed cell volume was measured using hematocrit capillary tubes. To achieve this, cells incubated in DME medium were resuspended in PBS and then fixed with 10% formalin. The cell concentration was adjusted to 10^9^ cells/mL, and then 200 µL of the cell suspension was loaded into hematocrit capillary tubes. The capillary tubes were placed vertically overnight at room temperature to allow cell packing by gravity. The packed volume of the cells was measured by calculating the ratio of the length of the packed cell volume phase to the length of total volume phase. Statistical difference in relative packed cell volume was calculated by one-way analysis of variance with Bonferroni's multiple comparison test using Prism 6 (GraphPad software).

### Chemical and radiation sensitivity test

Cells were cultured in 2 mL of YPD medium overnight at 30°C, serially diluted (1 to 10^4^ -fold dilutions) with sterile distilled water, and spotted (3 µL) onto solid YPD medium containing the indicated concentrations of a diverse range of chemical reagents. Plates were further incubated at 30°C and photographed after 2–5 days. For the γ-irradiation and UV-irradiation sensitivity tests, the grown cells were serially diluted (1 to 10^4^ –fold dilutions), spotted onto YPD medium, and exposed to γ-radiation (1 kGy and 3 kGy doses) or UV light (intensities of 150 and 200 J/m^2^) and further incubated at 30°C. Digital pictures of the plates were taken after 1–3 days.

### TUNEL assay

To label the fragmented DNA by apoptotic cell death, TdT-mediated dUTP nick end labeling (TUNEL) assays were performed as previously described [50]. Each strain [WT (H99), the *cgp1*Δ mutant (YSB1632), and the *cgp1*Δ::*CGP1* complemented strain (+*CGP1*, YSB3332)] was grown at 30°C in liquid YPD medium for 16 h, sub-cultured into fresh liquid YPD medium, and further incubated at 30°C until the cell density approximately reached an OD_600_ of 0.8 followed by treatment of 100 mM hydroxyurea for 3 h at 30°C. Cells were fixed, permeabilized, and then incubated with TUNEL reaction mixture (*In Situ* Cell Death Detection Kit, Roche) for 60 min at 37°C in dark and humid conditions. Cells were washed with PBS and labeled with Hoechst to stain DNA for 30 min at room temperature in dark. Hoechst and TUNEL stained cells were visualized by fluorescence microscope (Nikon, eclipse Ti). The mean GFP intensity was measured by fluorescence plate reader (Victor X5, PerkinElmer) from three biological replicates.

### Virulence assay

The wax moth *Galleria mellonella* was used as an insect model for *C. neoformans* virulence. Randomly selected wax moths at the final instar larval stage (body weight: 250 ± 50 mg, Vanderhorst Inc.) were divided into 5 groups (15 moths per group). Each *C. neoformans* strain [wild type strain (H99), *cgp1*Δ mutants (YSB1631 and YSB1632), the complemented strain of *CGP1* (YSB3332)] was grown overnight at 30°C in liquid YPD medium, washed three times with PBS, and resuspended in PBS. Cell concentrations were adjusted to 10^6^ cell/mL by using a hemocytometer and 4,000 cells were inoculated through the second to last prolegs of the larvae using a 100 μL Hamilton syringe with a 10 μL sized needle and a repeating dispenser (PB600-1, Hamilton). PBS was injected as the non-infection control. The injected insects were placed in Petri dishes in a humidified chamber and incubated in a 30°C incubator. The survival of the larvae was checked daily for up to 12 days. Larvae showing no movement upon touching were considered dead. Larvae transforming into pupae were censored for statistical analysis. Prism 6 (GraphPad) was used to create survival curves and perform statistical analysis using the Log-Rank (Mantel-Cox) test.

### Mating and cell fusion assay

*C. neoformans* strains of the opposite mating type (*MATα* and *MAT***a**) were incubated overnight in liquid YPD medium at 30°C, washed twice with distilled water and suspended to a final concentration of 10^7^ cells/mL. Equal volumes (5 μL) of the opposite mating type cells were mixed, spotted or streaked onto V8 agar medium, and incubated at room temperature in the dark for 1–2 weeks. Filament formation was monitored using light microscopy (Olympus BX51 microscope). Basidium and clamp connection was visualized by fluorescence microscope (Nikon, eclipse Ti). For cell fusion assays, opposite mating type cells were spotted onto V8 agar medium and were incubated for 24 hr at room temperature in the dark, harvested by scraping, resuspended in 1 mL of PBS, and 20 µL of the cell suspension was plated on YPD medium containing both nourseothricin and G418. The number of colonies on each plate was counted after incubation at 30°C for 3 days.

## Supplementary Material

1423189.zip

1423189.zip
