# Supplementary material for: The novel microtubule-associated CAP-glycine protein Cgp1 governs growth, differentiation, and virulence of Cryptococcus neoformans
Source: Virulence. 2018 Feb 27;9(1):566–84. doi: 10.1080/21505594.2017.1423189 (PMC5955475; doi:10.1080/21505594.2017.1423189)
Supplement: 1423189.zip [file kvir-09-01-1423189-s001.zip › 1423189/2017VIRULENCE0159R1-file002.docx]

**Table 1. Percentage of cells showing each number of nuclei**

| **% of cells** | | 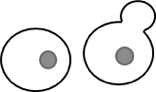  N | 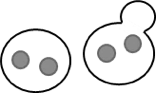  2N | 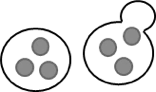  3N |
| --- | --- | --- | --- | --- |
| Strain | Condition |  |  |  |
| WT | Basal | 97 | 3 | 0 |
| WT | Benomyl treatment for 90 min | 90 | 8 | 2 |
| WT | TBZ treatment for 90 min | 92 | 6 | 2 |
| *cgp1*Δ | Basal | 74 | 22 | 4 |
| *cgp1*Δ | Benomyl treatment for 90 min | 84 | 11 | 5 |
| *cgp1*Δ | TBZ treatment for 90 min | 78 | 21 | 1 |
