# Supplementary material for: The novel microtubule-associated CAP-glycine protein Cgp1 governs growth, differentiation, and virulence of Cryptococcus neoformans
Source: Virulence. 2018 Feb 27;9(1):566–84. doi: 10.1080/21505594.2017.1423189 (PMC5955475; doi:10.1080/21505594.2017.1423189)
Supplement: 1423189.zip [file kvir-09-01-1423189-s001.zip › 1423189/2017VIRULENCE0159R1-s02.docx]

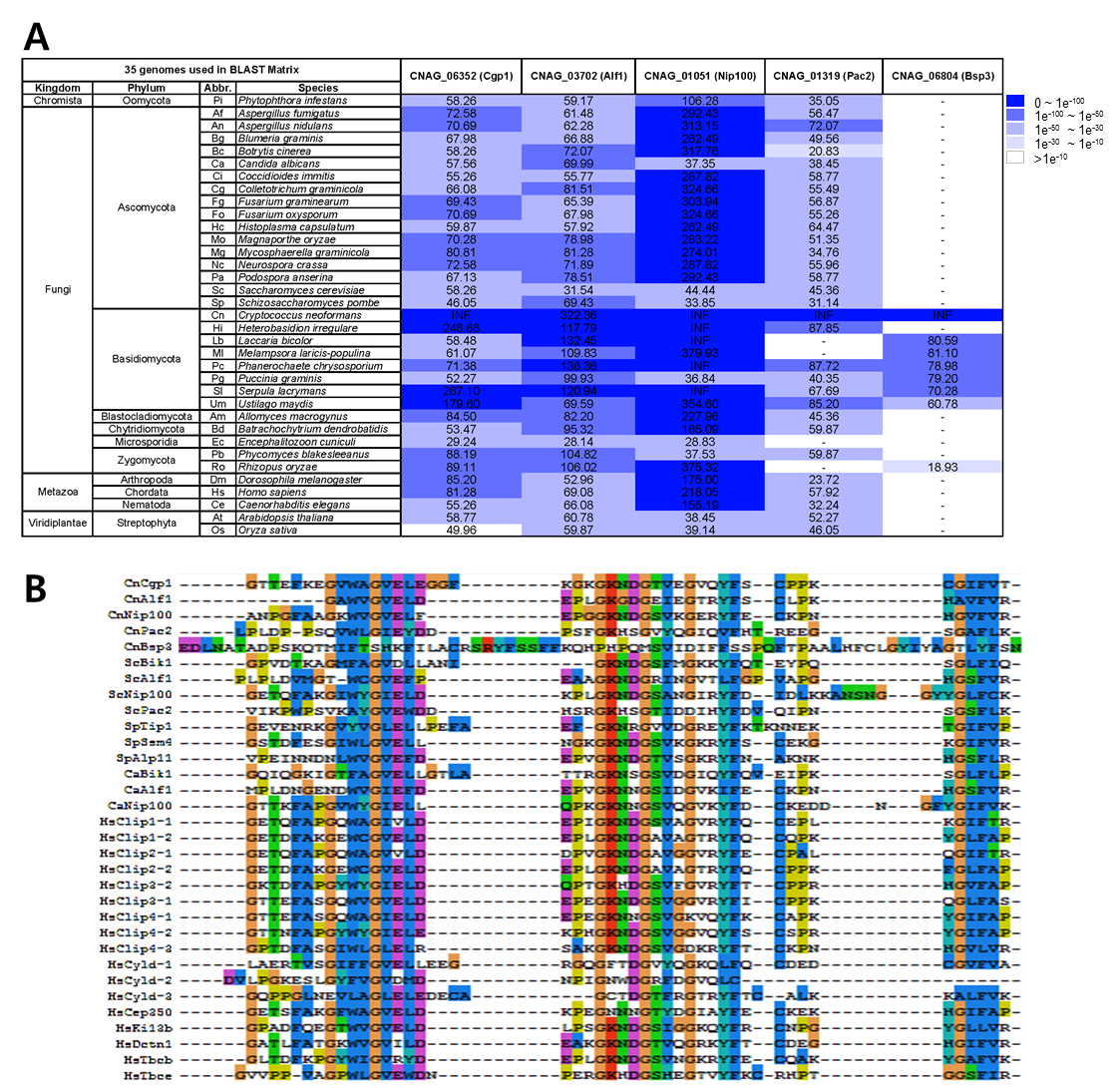


**Figure S1.** Conservation analysis of the CAP-Gly proteins and the Cpg1 CAP-Gly domain across species. (A) BLAST matrix for the five CAP-Gly proteins identified in *C. neoformans* using the Comparative Fungal Genomics Platform (CFGP, http://cfgp.riceblast.snu.ac.kr) database. Using the protein sequence queries, orthologous proteins were matched and retrieved from the genome databases from 35 eukaryotic species shown. (B) BLAST comparison of CAP-Gly domain sequences between *C. neoformans* (Cpg1) and 34 other species. We performed the multiple sequence alignment using ClustalX2 windows interface program run by University College Dublin. Searching for CAP-Gly proteins and each domain sequence was obtained using the UniProtKB database ([http://Uniport.org](http://uniport.org/)). Cn, *Cryptococcus neoformans*; Sc, *Saccharomyces cerevisiae*; Sp, *Schizosaccharomyces pombe*; Ca, *Candida albicans*; Hs, *Homo sapiens*.


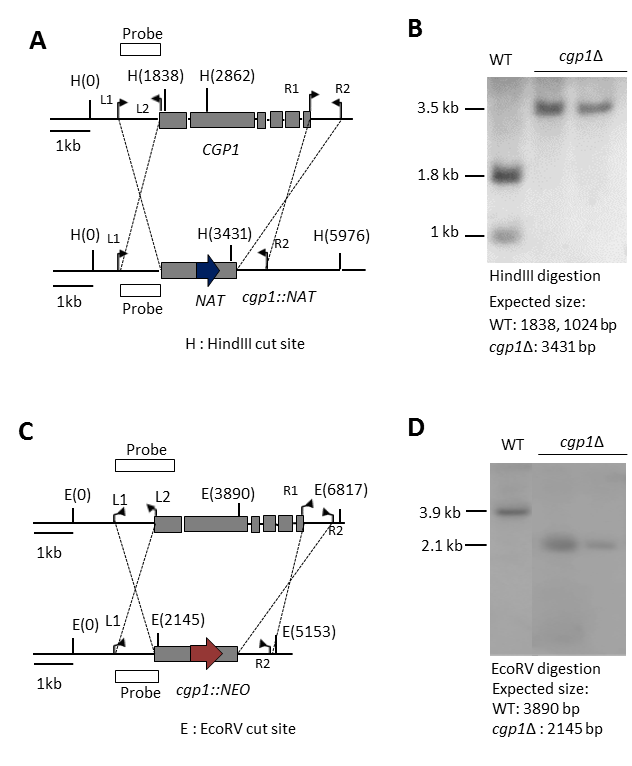


**Figure S2.** Disruption of *C. neoformans* serotype A *CGP1*. (A and C) Schematic of the disruption of the *CGP1* gene in serotype A *MAT*α H99 and *MAT***a** KN99 strain, respectively. Primers for the gene deletion cassettes are indicated as bent arrows. The probes used for Southern blot analysis are shown above or below the target genes. The *CGP1* gene was replaced with a nourseothricin-resistance gene (*NAT*^r^) or the neomycin-resistance gene (*NEO*^r^), respectively. (B and D) The correct genotype of the *cgp1*∆ mutants was verified by Southern blot analysis using genomic DNA digested with the restriction enzyme HindIII or EcoRV, respectively.


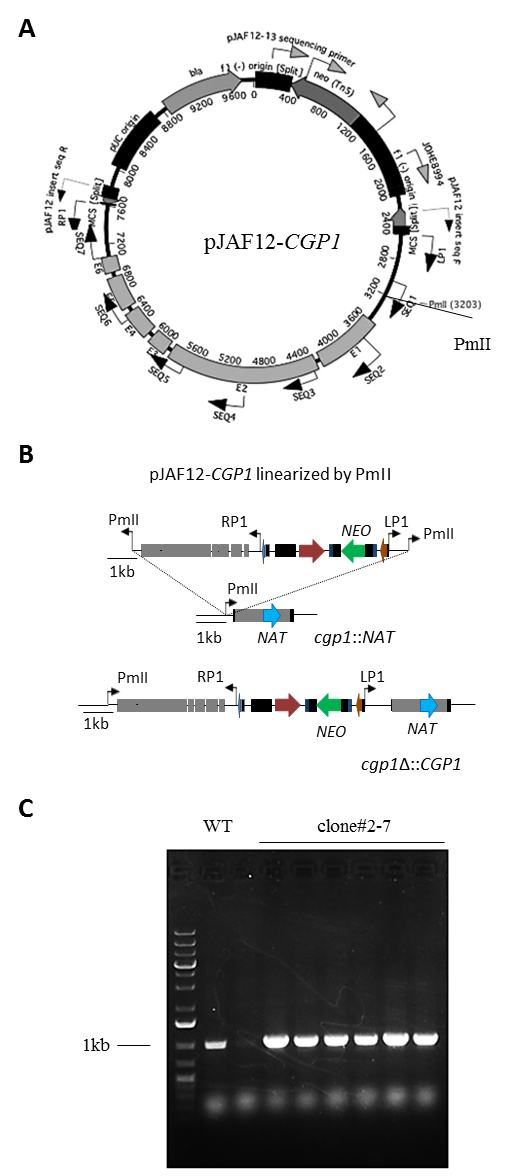


**Figure S3.** Construction of *cgp1*∆*::CGP1* complemented strains. (A) The cloned full-length *CGP1* gene containing the promoter, the terminator, and the open reading frame (ORF) was sub-cloned into plasmid pJAF12 to produce plasmid pJAF12-*CGP1*. (B) pJAF12-*CGP1* was linearized using restriction enzyme PmlI and was re-integrated into the native *CGP1* locus of the *cgp1*∆ mutant (YSB1632). (C) Diagnostic PCR used to confirm correct targeted reintegration of the *CGP1* gene. The primers B3713 and B6104 were for the diagnostic PCR analysis.


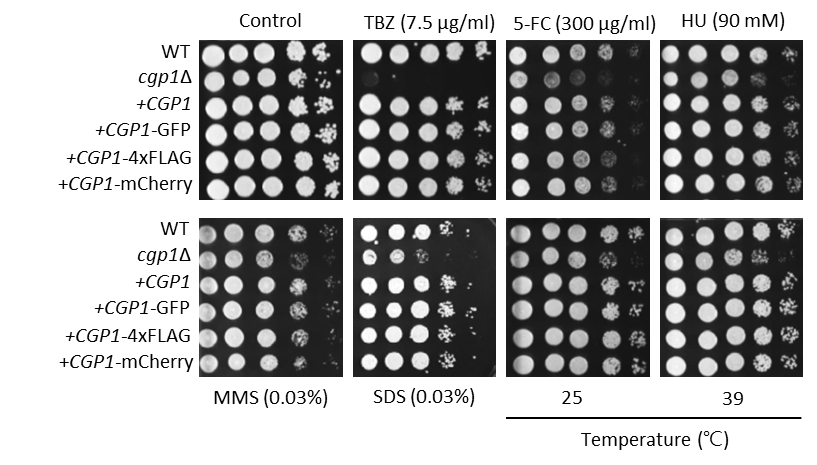


**Figure S4.** *cgp1*Δ::*CGP1*-GFP, *cgp1*Δ::*CGP1*-4xFLAG, and *cgp1*Δ::*CGP1*-mCherry strains exhibited wild-type levels of resistance in response to genotoxic DNA damage stress, cell membrane stress and high temperature. Strains [WT (H99), *cgp1*Δ mutant (YSB1632), +*CGP1* (*cgp1*Δ*::CGP1*, YSB3332), +*CGP1*-GFP (*cgp1*Δ*::CGP1*-GFP, YSB3358), +*CGP1*-4xFLAG (*cgp1*Δ*::CGP1*-4xFLAG, YSB3541) and +*CGP1*-mCherry (*cgp1*Δ*::CGP1*-mCherry, YSB3964)] were grown in a liquid YPD medium at 30^o^C overnight. Cells were 10-fold diluted (1-10^4^ fold dilutions) and then spotted onto the YPD medium containing the indicated concentration of stress inducers. The plates were incubated at 30^o^C or at the designated temperature and photographed 2-4 days later.

**
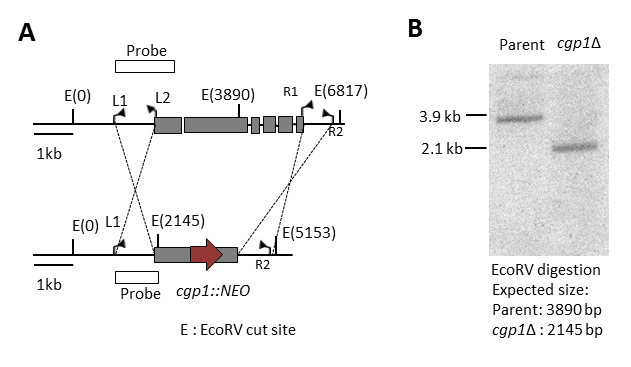
**

**Figure S5**. Construction of *cgp1*Δ *GFP-TUB* strain. (A) Diagrams for disruption of *CGP1* gene in GFP-Tubulin strain (LK126 as a parent). Primers for the gene deletion cassettes are indicated as bent arrows. Probe for Southern blot analysis was depicted above or below of target genes. The *CGP1* gene is replaced with neomycin-resistant gene (*NEO*^r^). (B) The correct genotype of the *cgp1*∆ *GFP-TUB* mutant (YSB5536) was verified by Southern blot analysis using genomic DNA digested with the restriction enzyme EcoRV.

**
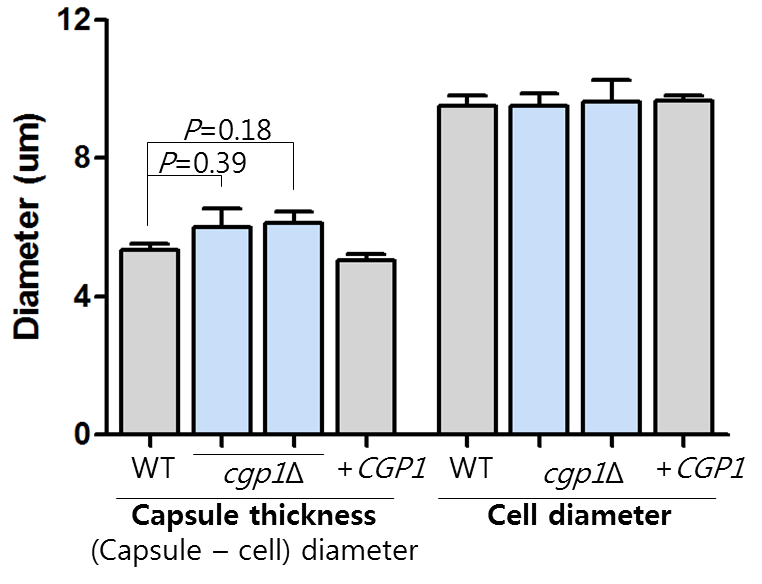
**

**Figure S6**. Measurement of capsule thickness and diameter. [WT (H99), *cgp1*Δ mutants (YSB1631 and YSB1632), and the *cgp1*Δ::*CGP1* complemented strain (+*CGP1*, YSB3332)] was grown overnight and then spotted onto solid DME agar medium and further incubated at 37°C for 2 days. Cells were fixed, stained by India ink, and observed by microscope. The diameter was measured by SPOT advanced software (ver. 4.6) and *P* value was calculated using the Student’s *t*-test by GraphPad Prism 5. Three-independent experiments were performed, and error bar indicates standard error of the mean (SEM).


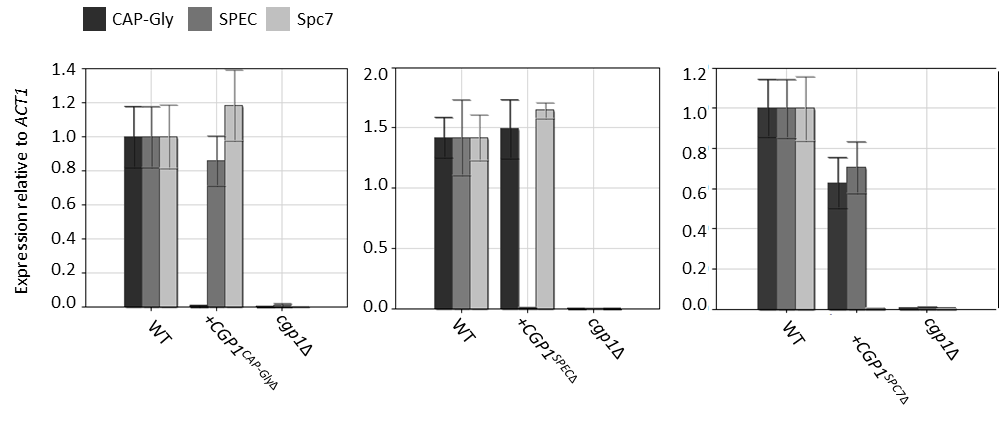


**Figure S7.** Measurement of the expression levels of *CGP1* domain deletion mutants. The expression levels of the truncated *CGP1* genes were quantified using RT-PCR analysis. The primers used for the CAP-Gly, the SPEC, and the Spc7 domains are listed in Table S1. Strains [WT (H99), *cgp1*Δ mutant, *cgp1*Δ::*CGP1^CAP-Gly^*^Δ^ (+*CGP1^CAP-Gly^*^Δ^), *cgp1*Δ::*CGP1^SPEC^*^Δ^ (+*CGP1^SPEC^*^Δ^), *cgp1*Δ::*CGP1*^SPC7Δ^ (+*CGP1^SPC7^*^Δ^)] were cultured in 50 mL of YPD medium for 16 h. A portion of the cell culture medium was inoculated into a fresh 50 mL of YPD medium and grown to OD_600_=1.0. Total RNA was isolated using Trizol reagent and cDNA was then synthesized from the total RNA as a template using reverse transcriptase. The *cgp1*Δ mutant and wild-type strain were used as negative or positive controls, respectively. The expression levels of *CGP1* gene was normalized to that of *ACT1*. The experiments were performed with two-independent biological replicates in two-independent mutants. Error bars represent standard error of the mean S.E.M.


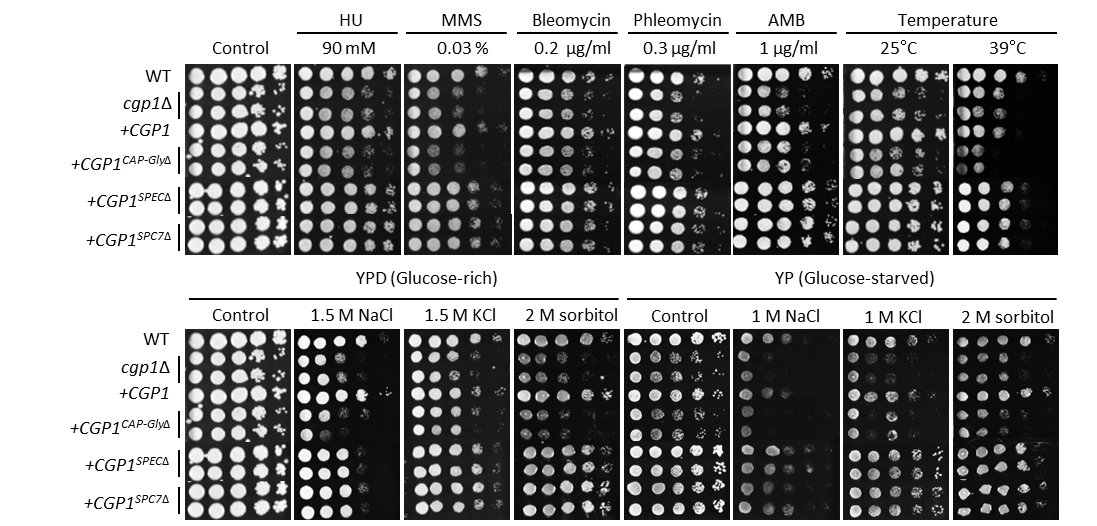


**Figure S8.** The CAP-Gly domain of Cgp1 is required for stress responses. Cells from each strain were cultured in YPD liquid medium for 16 h at 30°C. Cells were serially diluted (1-10^4^ fold dilutions) and spotted (3 μL) onto YPD solid medium containing the indicated concentration of stress reagents. Plates were further incubated and photographed after three days. Strain information: WT (H99), *cgp1*Δ mutants (YSB1631 and YSB1632), +*CGP1* (*cgp1*Δ*::CGP1*, YSB3332), *cgp1*Δ::*CGP1^CAP-Gly^*^Δ^ (+*CGP1^CAP-Gly^*^Δ^, YSB3897 and YSB3901), *cgp1*Δ::*CGP1^SPEC^*^Δ^ (+*CGP1^SPEC^*^Δ^, YSB3949 and YSB3957), *cgp1*Δ::*CGP1^SPC7^*^Δ^ (+*CGP1^SPC7^*^Δ^, YSB3905 and YSB3907).


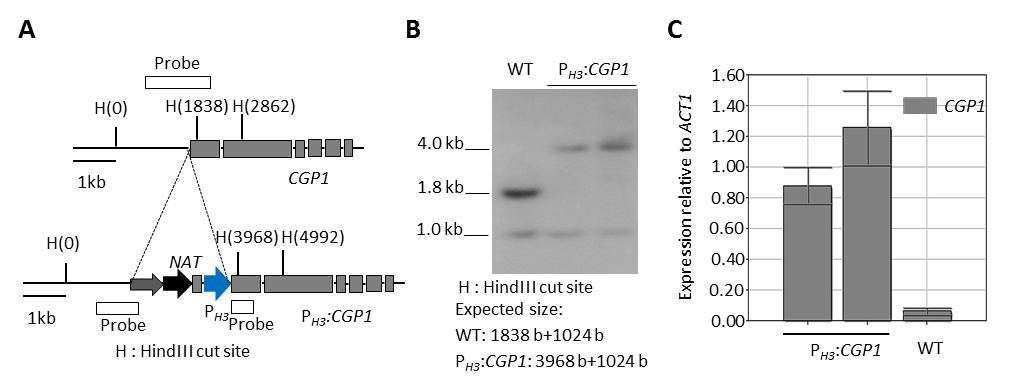


**Figure S9.** Construction of the *CGP1* overexpression strains. (A) Schematic showing the process for construction of *CGP1* overexpression strains though replacing the native promoter with the histone H3 gene promoter. (B) The correct genotype of the *CGP1* overexpression strains (P*_H3_:CGP1*; YSB3663 and YSB3665) were verified by Southern blot analysis of genomic DNA digested with the restriction enzyme HindIII. (C) Constitutive overexpression of *CGP1* was confirmed by quantitative RT-PCR analysis of two-independent biological replicates with the gene specific primers listed in Table S2. Error bar indicates SEM.


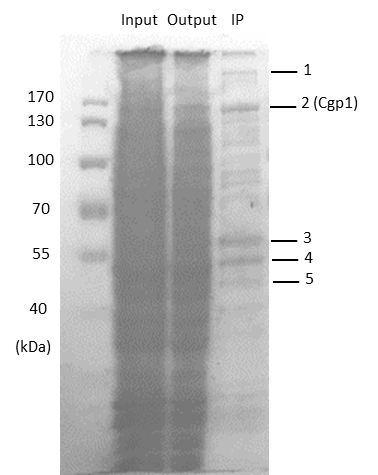


**Figure S10.** Identification of Cgp-interaction proteins using an *in vitro* pull down analysis. The *cgp1*Δ*::CGP1*-4XFLAG strain (YSB3541) was cultured in 50 mL of YPD medium at 30^o^C overnight. After this 5 mL of the cell culture was inoculated into 1 L of fresh YPD medium and further cultured for 4 hr. Total protein extracts were treated with an anti-FLAG antibody and then incubated overnight at 4^o^C. The protein extracts were treated with anti-FLAG antibody (Sigma-Aldrich, F1804) and further incubated with Protein G Sepharose 4 Fast Flow (GE, 17-0618-01) for 8 hr. The extraction was pelleted, washed, and resuspended in lysis buffer. The protein gel was stained using Coomassie brilliant blue and bands marked with number were subjected to LC-MS/MS analysis (Table S3). Input indicates the whole cell lysate and output represents the lysate after depletion with the anti-FLAG antibody.

**Table S1.** Strains used in this study

| Strain | Genotype | Parent | Reference |
| --- | --- | --- | --- |
| H99 | *MAT*α (Serotype A) |  | ^1^ |
| KN99**a** | *MAT***a** (Serotype A) |  | ^2^ |
| YSB1631 | *MAT*α *cgp1*Δ*::NAT-STM #228* | H99 | This study |
| YSB1632 | *MAT*α *cgp1*Δ*::NAT-STM #228* | H99 | This study |
| YSB3332 | *MAT*α *cgp1*Δ*::CGP1-NEO* | YSB1632 | This study |
| YSB3358 | *MAT*α *cgp1*Δ*::CGP1-GFP-NEO* | YSB1632 | This study |
| YSB3541 | *MAT*α *cgp1*Δ*::CGP1-4XFLAG-NEO* | YSB1632 | This study |
| YSB3889 | *MAT***a** *cgp1*Δ*::NEO* | KN99**a** | This study |
| YSB3891 | *MAT***a** *cgp1*Δ*::NEO* | KN99**a** | This study |
| YSB3897 | *MAT*α *cgp1*Δ*::CGP1^CAP-Gly^*^Δ^ | YSB1632 | This study |
| YSB3901 | *MAT*α *cgp1*Δ*::CGP1^CAP-Gly^*^Δ^ | YSB1632 | This study |
| YSB3949 | *MAT*α *cgp1*Δ*::CGP1^SPEC^*^Δ^ | YSB1632 | This study |
| YSB3957 | *MAT*α *cgp1*Δ*::CGP1^SPEC^*^Δ^ | YSB1632 | This study |
| YSB3905 | *MAT*α *cgp1*Δ*::CGP1^SPC7^*^Δ^ | YSB1632 | This study |
| YSB3907 | *MAT*α *cgp1*Δ*::CGP1 ^SPC7^*^Δ^ | YSB1632 | This study |
| YSB3968 | *MAT*α *cgp1*Δ*::CGP1-mCherry-HYG* | YSB1632 | This study |
| YSB3663 | *MAT*α P*_H3_:CGP1-NAT* | H99 | This study |
| YSB3665 | *MAT*α P*_H3_:CGP1-NAT* | H99 | This study |
| YSB119 | *MAT*α *aca1*Δ*::NAT-STM#43 ura5 ACA1-URA5* | H99 | ^3^ |
| YSB121 | *MAT***a** *aca1*Δ*::NEO ura5 ACA1-URA5* | KN99**a** | ^3^ |
| LK126 | *MAT*α *GFP-TUB::NAT* | H99 | ^4^ |
| YSB5536 | *MAT*α *GFP-TUB::NAT cgp1*Δ*::NEO* | LK126 | This study |

**Table S2.** Primers used in this study

| Name | Sequence (5' to 3') | Comments |
| --- | --- | --- |
| B79 | TGTGGATGCTGGCGGAGGATA | Common screening primer |
| B1026 | GTAAAACGACGGCCAGTGAGC | M13 forward extended primer |
| B1027 | CAGGAAACAGCTATGACCATG | M13 reverse extended primer |
| B1454 | AAGGTGTTCCCCGACGACGAATCG | *NAT*-split marker primer 1 |
| B1455 | AACTCCGTCGCGAGCCCCATCAAC | *NAT*-split marker primer 2 |
| B1886 | TGGAAGAGATGGATGTGC | *NEO*-split marker primer 1 |
| B1887 | ATTGTCTGTTGTGCCCAG | *NEO*-split marker primer 2 |
| B7056 | CGAGAAGAGCGAAATCGTC | *ALF1* screening primer |
| B7110 | TTAGCCAACCCATCTTCC | *ALF1* southern probing primer |
| B7057 | CAGAAGAATCAACCATCGC | L1 primer for 5’-flanking region of *ALF1* |
| B7058 | TCACTGGCCGTCGTTTTACCAAAGACACTGAGGTAAGGC | L2 primer for 5’-flanking region of *ALF1* |
| B7059 | CATGGTCATAGCTGTTTCCTGACATCTTTTCCGACGACG | R1 primer for 3’-flanking region of *ALF1* |
| B7060 | TCCCCTTTTGCTTCCAAC | R2 primer for 3’-flanking region of *ALF1* |
| B3712 | TGGTCTGGCAAGAAAGTG | *CGP1* screening primer |
| B3713 | CTGTAGGAACCCACTTTGC | *CGP1* southern probing primer |
| B3714 | GAGCAGCAAAGATTTCGC | L1 primer for 5’-flanking region of *CGP1* |
| B3715 | TCACTGGCCGTCGTTTTACGAGAGTTGAGGTAAGCGTGAC | L2 primer for 5’-flanking region of *CGP1* |
| B3716 | CATGGTCATAGCTGTTTCCTGTCTCATCTCGTGTTCCTACC | R1 primer for 3’-flanking region of *CGP1* |
| B3717 | GTCCTTCCCCTTTTTATGG | R2 primer for 3’-flanking region of *CGP1* |
| B6102 | CGGGCGGCCGCGAGCAGCAAAGATTTCGC | LP1 for *CGP1* complemented strain |
| B6103 | CGGGCGGCCGCCTGACTATAACCGGTACCTT | RP1 for *CGP1* complemented strain |
| B6104 | TCTTCAGGGGTTCTGTGTG | *CGP1* sequencing primer 1 |
| B6105 | CTACGAAGCAATGACTCCC | *CGP1* sequencing primer 2 |
| B6106 | AAGTTGTCGCCTCCCACTAC | *CGP1* sequencing primer 3 |
| B6107 | TGAGACAAGAGATGGAGCG | *CGP1* sequencing primer 4 |
| B6108 | AGTCAAGGATGGGGAGAAG | *CGP1* sequencing primer 5 |
| B6109 | TGCCCAAGGTAATCAGAAAG | *CGP1* sequencing primer 6 |
| B6110 | TGCTGTCTCTCGTGAACAAG | *CGP1* sequencing primer 7 |
| B6121 | CGGGCGGCCGCAAATACGTCTTCCGCCATGGGGCA | RP for Cgp1 tagging construction |
| B6859 | CGGCCCGGGGAGCAGCAAAGATTTCGC | *CGP1* domain deletion primer L1 |
| B6852 | CGGGCGGCCGCCTGACTATAACCGGTACCTT | *CGP1* domain deletion primer R2 |
| B6854 | CGGGGATCCGCTAGCTGTAGGAACCCACTTTGC | L2 for CAP-Gly domain deletion |
| B6853 | CGGGCTAGCCTCCCACTACCGGCACTT | R1 for CAP-Gly domain deletion |
| B6855 | CGGGCTAGCTTGCCTCCGTCCCCTTCT | L2 for SPEC domain deletion |
| B6856 | CGGGGATCCGCTAGCCGCAGTCAGAACGCGATT | R1 for SPEC domain deletion |
| B6857 | CGGGCTAGCGTTTCATTGATGAAAGAG | L2 for Spc7 domain deletion |
| B6858 | CGGGGATCCGCTAGCGGTATTTGCGGCTTGCAA | R1 for Spc7 domain deletion |
| B7024 | GAGTAAGAATCAGTAGTATG | LP for CAP domain qRT PCR |
| B7025 | CCTCCCTCAAGCTCAACGCC | RP for CAP domain qRT PCR |
| B7026 | GAGGATGCCCGAGTTACGGG | LP for SPEC domain qRT PCR |
| B7027 | CTGGGCCTTCAAAAACTCCA | RP for SPEC domain qRT PCR |
| B7028 | AGGAACTCAAAACAAAGCTT | LP for Spc7 domain qRT PCR |
| B7029 | GAAGCAATCTTGGCCTCCAA | RP for Spc7 domain qRT PCR |
| B679 | CGCCCTTGCTCCTTCTTCTATG | LP for *ACT1* qRT PCR |
| B680 | GACTCGTCGTATTCGCTCTTCG | RP for *ACT1* qRT PCR |
| B6652 | CACTCGAATCCTGCATGCTTAATGAGATTACAAGGTA | L2 primer for *CGP1* overexpression |
| B6653 | ACCACAACACATCTATCACATGTCACGCTTACCTCAAC | R1 primer for *CGP1* overexpression |
| B6654 | GAGATGAGGCAACAGAAGC | R2 primer for *CGP1* overexpression |
| B7063 | GCCAGCATCCACATACATC | SEQ2 for sequenceP*_H3_*:*CGP1 (NAT)* |
| B7064 | TCTTGACGACACGGCTTAC | SEQ3 for sequenceP*_H3_*:*CGP1 (NAT)* |
| B7065 | CAATACCATCCTTCCCACC | SEQ4 for sequenceP*_H3_*:*CGP1 (NAT)* |
| B7066 | AACAACAGGTATCCCAACG | SEQ5 for sequenceP*_H3_*:*CGP1 (NAT)* |
| B4017 | GCATGCAGGATTCGAGTG | H3 promoter-5’- flanking primer |
| B4018 | GTGATAGATGTGTTGTGGTG | H3 promoter-3’- flanking primer |
| B6380 | TCACTTGCATCTTCGACCAT | qRT PCR primer for *CGP1* |

**Table S3.** List of Cgp1-interacting proteins

| Regions  in Fig S8 | Gene locus  (CNAG_) | Size (KDa) | Score | Function |
| --- | --- | --- | --- | --- |
| 1 | 00520  02166 | 166.4  196.1 | 574.66  457.13 | Conserved hypothetical protein  DNA-directed RNA polymerase II largest subunit |
| 2 | 06352 | 114.1 | 420.38 | Cgp1 |
| 3 | 01727  01750  07445  05199  01404  00334 | 69.5  69.5  74.3  71.7  63.6  67.1 | 212.43  158.39  80.40  60.83  52.68  40.42 | Hsc70-4  Chaperone  Transketolase  Heat shock protein  Hsp71-like protein  Heat shock protein |
| 4 | 05750  07004  07676  03787 | 58.0  54.5  48.7  49.7 | 198.19  100.00  77.39  72.36 | ATPase alpha subunit  Lpd1  ATP-dependent RNA helicase Dbp2a  alpha tubulin |
| 5 | 06125  01413  00417  04028  03787  01840 | 50.3  46.0  46.7  39.4  49.7  50.0 | 217.37  215.63  133.41  118.82  35.52  34.61 | Translation elongation factor 1 alpha  Polyadenylated RNA-binding protein Pub1  Elongation factor 1 gamma  RNA binding protein  Alpha tubulin  Beta-tubulin |

**References**

1. Perfect JR, Ketabchi N, Cox GM, Ingram CW, Beiser CL. Karyotyping of *Cryptococcus neoformans* as an epidemiological tool. J Clin Microbiol 1993; 31:3305-9.

2. Nielsen K, Cox GM, Wang P, Toffaletti DL, Perfect JR, Heitman J. Sexual cycle of *Cryptococcus neoformans* var. *grubii* and virulence of congenic a and alpha isolates. Infect Immun 2003; 71:4831-41.

3. Bahn YS, Hicks JK, Giles SS, Cox GM, Heitman J. Adenylyl cyclase-associated protein Aca1 regulates virulence and differentiation of *Cryptococcus neoformans* via the cyclic AMP-protein kinase A cascade. Eukaryot Cell 2004; 3:1476-91.

4. Altamirano S, Fang D, Simmons C, Sridhar S, Wu P, Sanyal K, et al. Fluconazole-induced ploidy change in *Cryptococcus neoformans* results from the uncoupling of cell growth and nuclear division. mSphere 2017; 2.
