# Supplementary material for: The novel microtubule-associated CAP-glycine protein Cgp1 governs growth, differentiation, and virulence of Cryptococcus neoformans
Source: Virulence. 2018 Feb 27;9(1):566–84. doi: 10.1080/21505594.2017.1423189 (PMC5955475; doi:10.1080/21505594.2017.1423189)
Supplement: 1423189.zip [file kvir-09-01-1423189-s002.zip › 1423189/2017VIRULENCE0159R1-s01.doc]

Dec 21, 2017

Dear Dr. Eleftherios Mylonakis,

We thank you again for serving as the editor for our manuscript (2017VIRULENCE0159) entitled “**The novel microtubule-associated CAP-glycine protein Cgp1 governs growth, differentiation, and virulence of *Cryptococcus neoformans***”, for *Virulence* and for selecting four knowledgeable reviewers. We are pleased that all the reviewers found the revised manuscript satisfactory. We have revised the manuscript again according to reviewers’ editorial suggestions. For a single experimental comment made by reviewer 1, we made the quantification of the TUNEL assay result. Our point-by-point responses can be found in the attached letter.

The manuscript has been read and approved by all authors. None of the materials contained within the manuscript have been under consideration for publication elsewhere. We hope you find the 2^nd^ revised manuscript suitable for publication in *Virulence*.

Please feel free to contact us if you have any further questions.

Sincerely,

Yong-Sun Bahn, PhD

Professor

Dept. of Biotechnology, College of Life Science and Biotechnology, Yonsei University

50 Yonsei-ro, Seodaemun-gu, Seoul 03722, Korea

Email: ysbahn@yonsei.ac.kr
